# Tools for screening maternal mental health conditions in primary care settings in sub-Saharan Africa: systematic review

**DOI:** 10.3389/fpubh.2024.1321689

**Published:** 2024-09-26

**Authors:** Leveana Gyimah, Irene Akua Agyepong, David Owiredu, Elizabeth Awini, Linda Lucy Yevoo, Mary Eyram Ashinyo, Sorre Grace Emmanuelle Victoire Aye, Shazra Abbas, Anna Cronin de Chavez, Tolib Mirzoev, Anthony Danso-Appiah

**Affiliations:** ^1^Pantang Hospital, Accra, Ghana; ^2^Faculty of Psychiatry, Ghana College of Physicians and Surgeons, Accra, Ghana; ^3^Dodowa Health Research Centre, Research and Development Division, Ghana Health Service, Dodowa, Ghana; ^4^Faculty of Public Health, Ghana College of Physicians and Surgeons, Accra, Ghana; ^5^Department of Epidemiology and Disease Control, School of Public Health, University of Ghana, Legon, Ghana; ^6^Centre for Evidence Synthesis and Policy, University of Ghana, Accra, Ghana; ^7^Institutional Care Division, Ghana Health Service, Accra, Ghana; ^8^Nossal Institute for Global Health, Melbourne School of Population and Global Health, The University of Melbourne, Melbourne, VIC, Australia; ^9^Department of Global Health and Development, London School of Hygiene and Tropical Medicine, London, United Kingdom

**Keywords:** screening tools, diagnostic tools, maternal mental health, mental disorders, mental conditions, pregnant women, postpartum women, primary care

## Abstract

**Introduction:**

In sub-Saharan Africa, pregnant and postpartum women with mental health problems are often missed in healthcare systems. To address this, a practical and simple screening tool for maternal mental health should be available to primary healthcare workers. An important step toward having such a tool is to assess the existing tools and their effectiveness in primary care settings.

**Methods:**

We systematically searched PubMed, LILAC, CINAHL, Google Scholar, African Index Medicus, HINARI, and African Journals Online from inception to 31 January 2023, without language restriction. Reference lists of retrieved articles were reviewed and experts in the field were contacted for studies not captured by our searches. All retrieved records were collated in Endnote, de-duplicated, and exported to Rayyan for screening. Study selection and data extraction were done by at least two reviewers using a pre-tested flow chart and data extraction form. Disagreements between reviewers were resolved through discussion. We contacted primary authors for missing or insufficient information and conducted a content analysis of the psychometric properties of the tools.

**Results:**

In total, 1,181 studies were retrieved by our searches, of which 119 studies were included in this review. A total of 74 out of 119 studies (62%) were screened for depression during pregnancy and or the postpartum period. The Edinburg Postpartum Depression Scale (EPDS) and the Patient Health Questionnaire (PHQ-9) were the most commonly used tools. In total, 12 studies reported specificity and sensitivity for tools for measuring depression (EPDS, PHQ-9, and Whooley) and psychological distress [Self Report Questionnaire (SRQ) and Kessler Psychological Distress Scale (KPDS)]. The average sensitivity and specificity of the EPDS reported were 75.5 and 76.5%, respectively, at a cut-off of ≥13. The EPDS appears to be the most acceptable, adaptable, user-friendly, and effective in screening for maternal mental health conditions during pregnancy and postpartum. However, the methodological approach varied for a particular tool, and documentation on the attributes was scanty.

**Conclusion:**

The EPDS was the most commonly used tool and considered as most acceptable, adaptable, user-friendly, and effective. Information on the performance and psychometric properties of the vast majority of screening tools was limited.

**Systematic review registration:**

https://www.crd.york.ac.uk/prospero/display_record.php?ID=CRD42022323558, identifier CRD42022323558 (PROSPERO).

## Introduction

In 2018, several African countries were considered to be 4 years behind maternal, newborn, and child health targets for the attainment of the sustainable development goals (SDGs) (1). This has been further compromised by the effects of the COVID-19 pandemic (2). Although infant and child health are intricately linked to maternal health and wellbeing, most countries have prioritized the former over maternal health (3, 4). In particular, the mother’s mental health may affect the health status of their children (5–7), and thus addressing the mental wellbeing of mothers should be seen as key to improving both maternal and child health outcomes (8). Undoubtedly, the foundation for quality childhood development must begin with comprehensive programs that address maternal health and wellbeing (9–11). Therefore, in 2022, the WHO launched guidance for the integration of mental health services into primary care as the most viable way of narrowing the mental health gap in LIMICs, placing emphasis on maternal mental health (MMH) (12). However, key challenges for such integration have been the low capacity to detect and manage mental disorders by non-specialized primary care health workers and the lack of cross-culturally validated screening tools (13).

MMH conditions are psychiatric disorders that can occur before pregnancy, during pregnancy (antepartum), or after delivery (postpartum). A significant proportion of women experience these conditions during the perinatal period, which includes both the period during pregnancy and after delivery (14–18). The burden of MMH conditions varies among low-middle- and high-income countries due to multiple factors such as genetic predisposition, previous history of unfavorable birth outcome, and socio-economic status (19). Most common disorders, mainly anxiety and depression, affect an estimated 20% of women in low-income countries (17) and 10% in high-income countries (20, 21) during pregnancy and the postpartum period. Pregnancy increases the vulnerability of women, particularly their physiological and mental health, evidenced by their low capacity to cope with stress and normal daily activities (22). It is estimated that up to a third of women presenting with depressive episodes in the antepartum period have no previous history of depression (22). Pregnant women with pre-existing mental illness are at a higher risk of recurrence or exacerbation during the pregnancy and after delivery (12).

In Sub-Saharan Africa (SSA), several studies have reported a range of MMH conditions, commonly depression (10–70%) and anxiety (10–50%) during pregnancy and postpartum period (18, 23–25). These disorders have been linked with poverty, experience of adverse life events, intimate partner violence, and poor social support to name but a few (24–29). One of three postpartum mood disorders, postpartum depression (PPD) typically begins within 2 weeks after childbirth but can occur anytime within the first 12 months after delivery (30). Signs and symptoms of PPD may include persistent sadness, guilt, insomnia, anxiety, and thoughts of infant harm and self-harm (22, 30). Treatment is vital for PPD because symptoms can linger for years after childbirth if untreated (31).

Common mental disorders (CMDs) such as anxiety, depression, suicide and substance use, and psychosis can be debilitating as they affect thought patterns, feelings, behaviors, and relationships, as well as home and community functions (7, 15, 19, 24–26, 32–39) during pregnancy and postpartum periods (40–44). Globally, PPD is estimated to affect 10–15% of women within 4 weeks to 1 year after delivery (45–47). Evidence suggests PPD increases the risk of suicidal ideations in the mother (48) including potential harm (49–51) and poor health outcomes of the infant (52). Though initially thought to be less common in low- and middle-income countries (LMICs), current research shows that PPD is high in these countries (13, 47). The proportion of women with PPD was estimated to be 900,000 a year in 2015, but only 6% of these women reportedly would seek care (53). The low tendency to seek healthcare could be due to the lack of responsive health systems toward the needs of this vulnerable group (53, 54). The problem in LMICs is exacerbated by context, lack of social support, poverty, and negative life events such as the positive HIV status of the mother (55, 56).

Tools for screening MMH conditions encompass a variety of structured questionnaires or assessment protocols tailored to detecting conditions such as antenatal and PPD, anxiety disorders, and postpartum psychosis among pregnant and postpartum women. Globally, the Edinburg Postnatal Depression Scale, the 9-item-Patient Health Questionnaire (PHQ-9), the Centre for Epidemiological Studies-Depression Score (CES-D), the 20-WHO-Self Report Questionnaire (SRQ-20), the Kessler Psychological Distress Scale (K10), the General Anxiety Disorder and the Hopkins Symptom Check List (HSCL) are the common screening tools for assessing MMH conditions. The same tools are used in LMICs and SSA, with some adapted before use in SSA. Each screening tool inherently incorporates specific ‘screening’ criteria (symptom frequency and duration, cut-off scores) tailored to identify various MMH problems, such as depression and anxiety. Typically, these criteria are based on widely accepted standards, including those from the Diagnostic and Statistical Manual of Mental Disorders (DSM) and the International Classification of Diseases (ICD), as well as adaptations suitable for the cultural and healthcare context of SSA.

Screening for CMDs during the pregnancy and postpartum periods is not routinely done in most SSAs unless there is, perhaps, a personal or family history of a mental disorder (57, 58). Multiple screening tools have been documented, mainly in the context of research, for assessing MMH conditions (59) but their potential for routine use in primary care does not appear to be well-researched. In most African countries, screening tools developed in Western countries are usually translated into the local language and used without necessarily assessing the impact of their psychometric properties in the local context (59, 60) despite the fact that the expression of mental health conditions is influenced by culture and context (61–65). There is paucity of data on the available tools and contexts in which they are used across SSA countries.

There are a number of existing systematic reviews on MMH screening tools and programs (66–71). However, these reviews either focused on the global context (66, 72), or specific country context (68), targeted one or few MMH conditions (chiefly, depression, and anxiety) (66, 67, 69–71, 73–76), or focused on an individual screening tool (69–71, 73–76). SSA is characterized by unique cultural, socioeconomic, and healthcare delivery challenges (77–79), impacting these diagnostic tools that are culturally and context-sensitive. Moreover, the focus of our review on primary care settings aligns with efforts to integrate mental health services into existing healthcare infrastructure in SSA, which is in line with the WHO’s Mental Health Gap Action Programme (mhGAP) (80) and the SDGs.

## Rationale

While the antenatal care (ANC) and skilled birth attendance (SBA) coverage have improved, it is largely unknown whether health professionals in primary care facilities have the necessary skills that enable them to screen for mental health conditions of pregnant and postpartum women visiting primary care facilities in SSA countries and what context-sensitive screening tools are available.

An important innovation to improve the responsiveness of primary healthcare systems in SSA with more whole person-centered health systems approaches to MMH is the development of a simple, context-relevant, inexpensive, and user-friendly mental health screening tool for frontline primary healthcare workers. In the first step toward the development of such a tool, it is important to identify tools currently available for detecting MMH conditions, their sensitivity and specificity, and their potential usefulness in the SSAn health systems using a systematic or scoping review.

Our review questions were as follows: What screening tools for MMH have been used and evaluated in SSA in the antepartum and/or postpartum period?; What is known about their sensitivity and specificity, and what is their potential for use or adaptation for use at the primary care level in Sub-Saharan African health systems? The overarching aim of this review was to collate and describe the available evidence on MMH screening tools used in primary care settings in SSA. Specifically, this systematic review documents available tools as reported in the published literature, their effectiveness, and assesses their potential for early detection of MMH distress as part of antenatal and postnatal care in primary healthcare facilities.

## Definitions

### Mental health, psychiatric disorders, and mental health conditions

Mental Health is a state of wellbeing in which every individual realizes his or her own potential can cope with the normal stresses of life, can work productively and fruitfully, and is able to make a contribution to his or her community (81).

A psychiatric disorder is a clinically significant behavioral or psychological syndrome or pattern that occurs in an individual and is associated with distress, disability, or impairment in one or more important areas of functioning.

Mental health conditions are a group of disorders characterized by significant disturbances in a person’s thoughts, emotions, or behaviors that impact their ability to function in daily life. These conditions cause considerable distress or impairment in social, occupational, or other important areas of functioning.

These definitions align with criteria used in diagnostic manuals which provide specific guidelines for diagnosing various mental health conditions (82, 83).

### Tools, scales, interviews, and schedules

Psychological tools is a general term for any instrument or method used in psychological assessment and treatment (84–86). It encompasses any instrument or method used to assess, evaluate, or treat psychological conditions. This includes scales, interviews, questionnaires, and various assessment tools. These have been elaborated as follows:

Psychological scales are standardized instruments that measure specific psychological attributes or symptoms using standardized questions and rating systems.

Psychological interviews are used to conduct detailed assessments through structured or semi-structured conversations which gather detailed information about the client’s mental state, life history, and psychological functioning. Interviews can be diagnostic, exploratory, or therapeutic.

Psychological schedules are systematic tools used to organize and structure the administration of assessments or interventions. They outline the sequence and content of questions or tasks to be administered.

### Reliability and validity

The concept of reliability and validity (84, 85) provides a foundation for understanding how well psychological tools function and how their results can be interpreted. Reliability refers to the consistency or stability of a measurement tool over time. A reliable tool produces consistent results when used under the same conditions. Validity refers to the extent to which a tool measures what it is intended to measure. A valid tool accurately reflects the construct it aims to assess.

### Sensitivity and specificity

Sensitivity refers to the ability of a test to correctly identify individuals who have a particular condition, while specificity refers to the ability of a test to correctly identify individuals who do not have a particular condition (87, 88). Sensitivity and specificity are statistical measures commonly used to assess the performance of a tool or test against a gold standard. Sensitivity and specificity are crucial metrics for evaluating the effectiveness of diagnostic tests, where sensitivity focuses on correctly identifying those with the condition, and specificity focuses on correctly identifying those without it.

### Effectiveness of tools

The effectiveness of tools is an overarching concept that integrates elements of reliability and validity, focusing on how well a tool performs its intended function in practical, real-world scenarios (87). The degree to which a tool successfully accomplishes its intended purpose or achieves its objectives in real-world settings. This includes how well it performs in terms of practical utility, impact on outcomes, and the extent to which it improves the target condition or behavior.

### Psychological tool development

Psychological tool development involves a systematic process of conceptualization, design, testing, and standardization. These tools are used across various settings for assessment, intervention, screening, and research purposes, depending on their intended application and the context in which they are used.

## Review methods

This systematic review was developed in line with an established review methods framework (89). A review protocol was developed and revised after feedback from healthcare providers, policymakers, patient groups, and experts involved in systematic review methods. We did not apply PICOS (P—participants/population; I—intervention; C—control; O—outcomes; and study types) in the strictest sense, rather we used a modified form PCC (P—participants/population, C—concept, and C—context) for defining our criteria for considering studies in this review since our review aimed to systematically explore the literature to identify key concepts, theories, sources of evidence, and gaps in research (90).

### Criteria for considering studies for inclusion in this review

#### Type of studies

Studies that reported a tool used for detecting or screening for any mental health condition in the pregnancy, delivery, and postpartum period in a primary clinical care setting in SSA and describing the psychometric properties of the tool were eligible for inclusion. Additionally, the study design had to be either of the following: a randomized controlled trial (RCT), cohort, case–control, and cross-sectional study or case series. Reviews, commentaries, case studies, or opinions were not eligible for inclusion. We went through the full text and reference lists of included studies to identify further potentially eligible studies missed by our searches. If the study was part of a global review having, for example, SSA as a sub-set or sub-regional focus such as an East/West/Central/Southern African focus, such a review was not included as a whole. Instead, we retrieved the studies conducted in the SSA primary care context for inclusion. If the study reported a country or regional estimate without a well-defined sample (representative sample or sub-sample within the source population), it was considered not eligible for inclusion. For multi-country studies that included studies from SSA and reported separately for each of the countries, data from the SSA context were selected for inclusion.

#### Population

Our population of interest was women in the pregnancy, delivery, and postpartum period. We defined pregnancy as the period from conception (starting from the last menstrual period) to delivery, lasting on average 40 weeks (22); and the postpartum or postnatal period as the period from birth of the baby to 12 months after delivery (45, 47). There are variations in the definition of the length of the postpartum period in the literature (45, 46, 91) and the choice of 12 months was based on the definition by the International Marce Society for perinatal mental health. We defined a primary care facility to include all places or facilities that provide perinatal care, including formal sector facilities such as hospitals or clinics, and informal facilities such as traditional and faith-based healers; as long as they reported on the use of a tool for detecting MMH condition in the SSA context. Polyclinics of tertiary facilities staffed with non-specialist mental health professionals were considered as primary care and were eligible for inclusion. Pregnant and postpartum women who sought care in a specialized tertiary facility, or by psychiatrists or specially trained (clinical) psychologists were not eligible for inclusion.

#### Concept

We considered diagnostic tools applied in an SSA context for screening or detecting MMH problems in pregnant and postpartum women who sought care in a primary care facility. The concepts explored included the availability of screening tools for screening MMH conditions, performance of tools (specificity and sensitivity as reported by the primary study investigators), and psychometric properties of the tools such as ease of use/application, preference, how it is understood by the patients, context relevance, type of health professionals who are able to apply it, overall performance of the tool, appropriateness, feasibility, and adaptability for primary care practice in Sub-Saharan African setting. Any other information or characteristics that further described the tools were considered.

Understandability of the screening tool refers to how the participants appreciate or comprehend what the questions on the screening tool are seeking to elicit and their capacity to provide an appropriate response based on the specific requirements of the tool. In addition to the specific questions stated, whether the options or the instruction for selecting an applicable response is appropriate for the specific target group. This is sometimes influenced by the culture and other factors of the participant and the context in which the questionnaire was originally developed. Ease of use of the screening tool refers to the user-friendly nature of the screening tool usually from the point of view of the administrator of the questionnaire. It therefore can be ranked by the participant for a self-administered questionnaire or by the interviewer for an interviewer-administered questionnaire. In some instances, the psychometric properties may be judged by the duration of administration, particularly in primary care settings, where staff may be overburdened by daily consultations. Adaptability of the screening tool refers to the ease or otherwise of altering aspects of the questionnaire to make it relevant to the target population ensuring the purpose of the tool is achieved. This may include changing the wording without losing the content of what it seeks to measure or translation into a locally understood language without losing the meaning of the questions participants are supposed to answer. Commonly, the tools are worded in the English language as they are developed in the global North.

#### Context

This systematic review considered the detection of MMH conditions in primary care facilities in all countries, cultures, and contexts across SSA. We explored regional differences in the availability and adaptability of tools for screening MMH conditions, grouping the regions into East, West, Central, and Southern Africa. We considered South Sudan as part of East Africa. We were interested in the types of MMH conditions identified in research as well as primary care delivery settings where pregnant and postpartum women received care.

#### Outcomes

##### Primary outcomes

- Availability of screening tools used in countries across SSA
- Performance of the tools (specificity, sensitivity, etc.) as reported in the primary studies

##### Secondary outcomes

- Proportion of MMH conditions identified by the tools across SSA
- Types of MMH conditions identified across countries in SSA

### Searches in electronic databases and other sources

We systematically searched the following electronic databases: PubMed, PsycINFO, LILAC, Google Scholar, and CINAHL from inception to 31 March 2023, without language restriction. In addition, we searched, African Index Medicus, HINARI, African Journals Online, IMSEAR, and Maternity and Infant Care (MIC) under MIDIRS and Global Health. The search terms (Supplementary Table S1) and search strategy (Supplementary Table S2) have been reported as Supplemental materials. Gray literature including dissertations, preprint repositories, and conference proceedings were searched; reference lists of retrieved articles were reviewed and experts in this field were contacted for studies that could not be captured by our searches, including unpublished studies.

### Managing the search results and selecting studies

All the studies retrieved from the electronic databases and gray literature were exported to Endnote (92), collated, and deduplicated. Then the articles were exported to Rayyan QCRI (93) for screening using a flow chart developed from the eligibility criteria (Supplementary Table S3). Two reviewers independently screened titles and abstracts of all retrieved articles for potentially eligible studies. Then, full texts were obtained for all the potentially eligible studies for further screening for inclusion in the review. Those that did not meet our eligibility criteria at the full-text stage were excluded with reasons for exclusion. Disagreements between screeners on the eligibility of studies were resolved through discussion between the reviewers.

### Data collection

Five trained data abstractors collected data using the data extraction form developed and pretested by the review team. Relevant psychometric properties and attributes on each tool that would enable establishing commonalities and differences across the included tools in terms of populations among which they have been used, and whether and in what ways they have been validated. To ensure reliability between reviewers, a series of training exercises was conducted prior to commencing screening. The data abstraction form was piloted on a random sample of 10 included articles and modified as required based on feedback from the team. Specifically, we extracted study characteristics such as the year in which the study was conducted, the year in which the study was published, the country in which the study was conducted, the study design used, and characteristics of participants such as age, level of education, socio-economic status, and occupation; obstetric factors (pregnant or postpartum); mental health conditions (PPD, postpartum anxiety, depression in pregnancy, and anxiety in pregnancy); and elements of the screening tools such as the name of the specific tool, whether the tool was applied to the mothers alone or in combination with others, which tool was used as the reference standard, and which tool was used as the index tool, and specificity and sensitivity, if reported. We extracted additional relevant information or attributes of the tool that could help with decisions to adopt the tool for use such as ease of use, and type of professionals required to administer the tool. We did not extract quantitative data such as the numbers of true positives, false positives, true negatives, and false negatives for calculating test accuracy, as this will be reported separately in a quantitative systematic review with meta-analysis. When necessary, we contacted the authors of the published articles of included studies to see if they could clarify or supplement the published results or provide raw data that we could use. There was a high level of consistency between the data extractors. Disagreements between data extractors were resolved through discussion.

### Data synthesis

The synthesis included simple quantitative analysis to generate frequencies, means, and ranges, where necessary. The qualitative synthesis focused on content analysis of mainly the psychometric properties of the tools, considering where the study was conducted (study setting) and the types of participants. Data were synthesized according to the research questions to identify similarities and differences, where relevant, as well as the patterns of the data similar to the process of reviewing heterogeneous studies in a meta-analysis. The reasons for the differences and similarities in results and the pattern of findings were reviewed. The overall synthesized studies were presented as tables under key headings. Where reported, sensitivity and specificity are summarized in Table 1.

**Table 1:** Sensitivity and specificity of screening tools as reported by the primary studies.

Study ID	Country	Study setting*	Sample size	Screening tool	MMH condition	Cut-off point	Sensitivity	Specificity
Abebe et al., 2019 (110)	Ethiopia	Primary care	511	EPDS	Postpartum depression	≥13	79%	75%
Abiodun, 2006 (103)	Nigeria	Primary care	360	1. EPDS 2. PSE	Postpartum depression	9 …	88% …	84% …
Chibanda et al., 2010 (108)	Zimbabwe	Primary care	210	EPDS (Shona version)	Postpartum depression	11/12	88%	87%
Nhiwatiwa et al., 1998 (99)	Zimbabwe	Primary care	500	SSQ	Postpartum Mental Disorder	≥8	82%	66%
Kimbui et al., 2018 (133)	Kenya	Primary care	212	1. EPDS 2. BDI-II 3. AUDIT	1. Depression 2. Substance use	≥8 … …	86% … …	73% … …
Nyamukoho et al., 2019 (175)	Zimbabwe	Primary care	197	EPDS	Depression	12	88%	87%
Chorwe-Sungani and Chipps, 2018 (124)	Malawi	Primary care clinics	97	1. EPDS 2. SRQ 3. HSCL-15 4. 3-item screener	Depression	≥10 ≥ 10 > 1.75 ≥ 1	88%, 72% 72% 97%	74% 96% 93% 88%
Rochat et al., 2013 (162)	South Africa	Primary care Clinic	112	1. EPDS 2. SCID	Depression	≥13 …	69% …	78% …
Adamu and Adinew, 2018 (111)	Ethiopia	Health Centre	629	EPDS	Postpartum Depression	≥13	79%	75%
Chibanda et al., 2014 (177)	Zimbabwe	Primary care clinics	210	1. EPDS 2. DSM-IV	Depression	≥11 …	88% …	87% …
Chorwe-Sungani and Chipps, 2018 (125)	Malawi	Antenatal clinics	480	1. EPDS 2. PRQ 3. MINI	Depression	≥10 ≥46	68% 44%	88% 92%
Cumbe et al., 2020 (144)	Mozambique	Primary care clinics	502	1. PHQ-9 2. PHQ-2 3. MINI	Depression	≥9 ≥2 …	72% 74.4% …	79% 71% …
Heyningen et al., 2018 (160)	South Africa	Primary care antenatal clinic	376	1. EPDS 2. EPDS-3A 3. PHQ-9 4. PHQ-2 5. KPDS-10 6. KPDS-6 7. Whooley 8. Whooley + help 9. GAD-2	1. Depression 2. Anxiety	13 3 10 2 11 8 2 2 2	75% 70% 66% 75% 80% 74% 66% 73% 64%	78% 77% 76% 69% 79% 85% 87% 82% 74%

EPDS, Edinburg Postnatal Depression Screen; PSES, Present State Examination Schedule; SSQ, Shona Symptom Questionnaire; AUDIT, Alcohol Use Disorder Identification Test; BDI-II, Becks Depression Inventory II. 3-item screener for depression. DSM-IV, Diagnostic and Statistical Manual of Mental Disorders (Fourth Edition); MINI, Mini International Neuropsychiatric Interview; PRQ, Pregnancy Risk Questionnaire; SCID, Structured Clinical Interview for Depression; HSCL-15, Hopkins Symptoms Checklist-15; SRQ, Self-Reporting Questionnaire; PHQ-9, Patient Health Questionnaire-9; PHQ-2, Patient Health Questionnaire-2; KPDS-10, Kessler Psychological Distress scale (K10); KPDS-6, Kessler Psychological Distress scale (K6); Sensitivity and specificity have been rounded to the nearest whole numbers; PSE, Present State Examination, is a clinical examination and no cut-off point for sensitivity provided. Kimbui et al. (133) has sensitivity and cu-off for only EPDS but not BDI-II and AUDIT.

### Dealing with missing data

We contacted the primary study authors for additional data or insufficient information. Where it was not possible to obtain the missing information, data were synthesized based on those with complete outcome data.

## Results

### Description of the included studies

**Figure 1:**
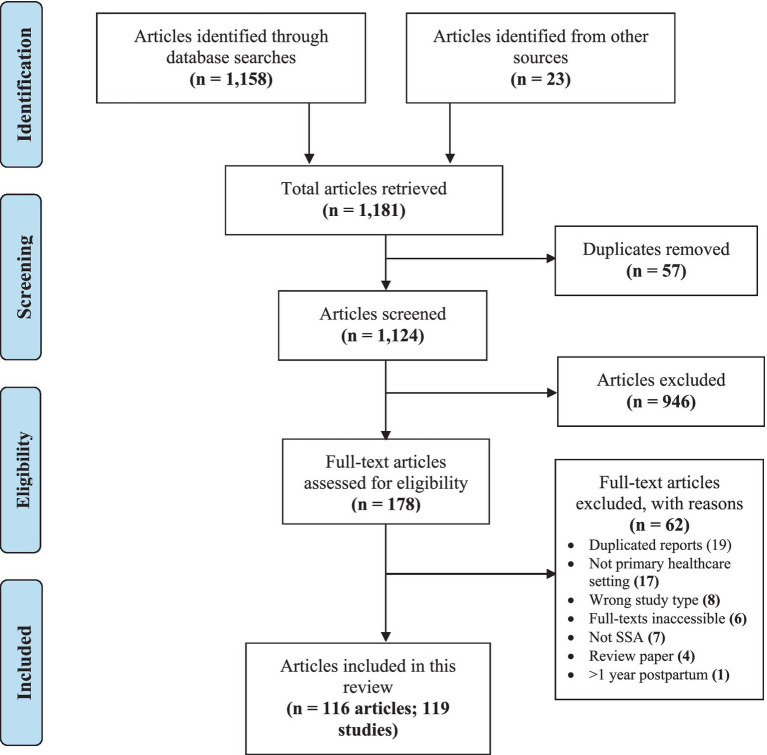
PRISMA flow diagram showing the study selection.

We retrieved 1,158 studies from electronic databases and 23 from other sources, making a total of 1,181 studies. After removing 57 duplicates, 1,124 studies were left, of which 946 were excluded following titles and abstracts screening. In total, 178 full-text documents were retrieved, and 62 records were excluded with reasons, leaving 116 papers. Three of these papers (94–96) presented data from multi-country studies (conducted in Ghana and Cote D’Ivoire in each case) which were disaggregated into six studies (Figure 1). The characteristics of the included studies are presented in Supplementary Table S4 and the full results in Supplementary Table S5.

In total, 116 papers from 119 studies met the inclusion criteria. Out of this number, three studies (97–99) were published before 2000; all three were published in the 1990s. A total of 11 (8.9%) (29, 100–109) were published between 2000 and 2010 and 114 (92.7%) were published after 2010 (Supplementary Table S4).

**Figure 2:**
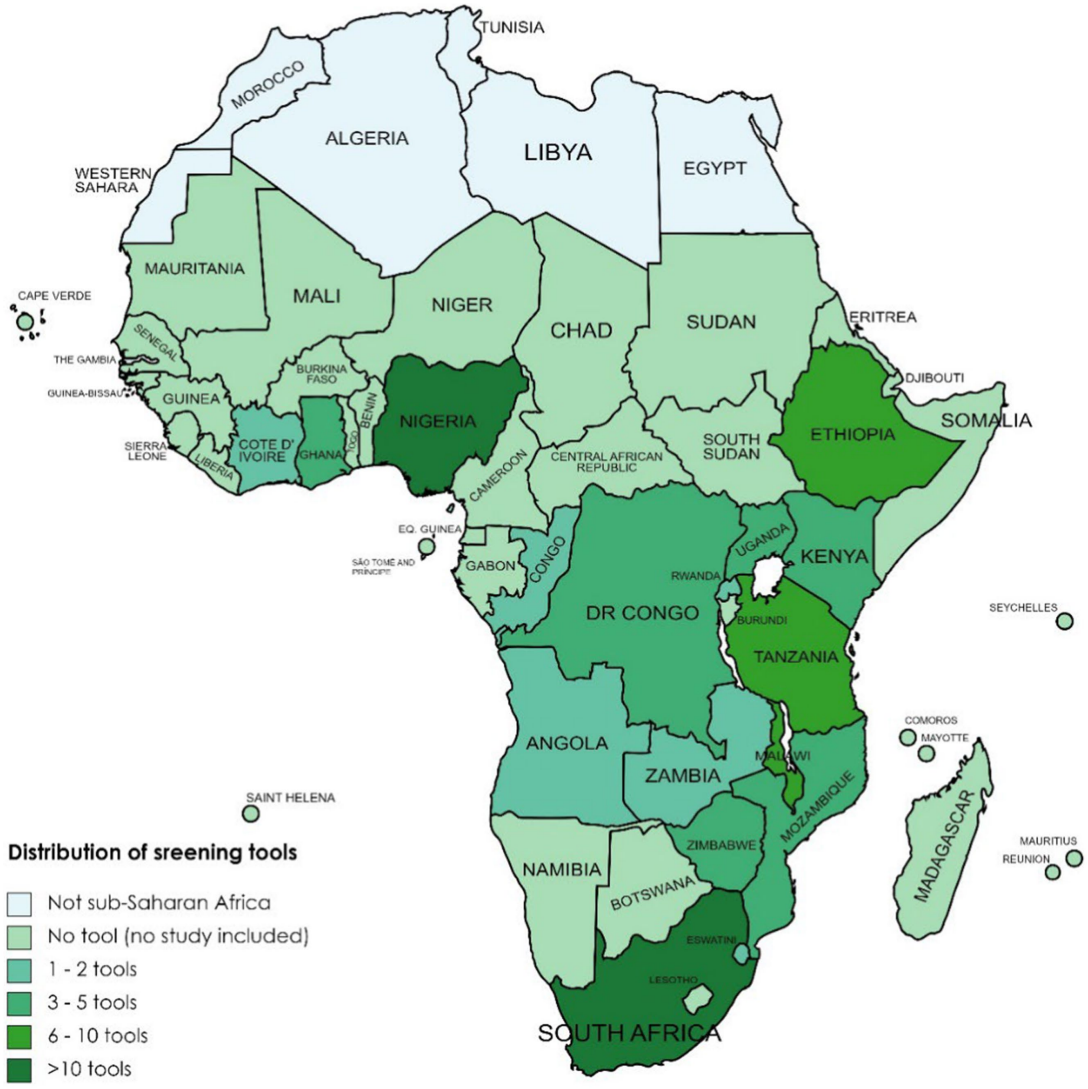
Availability and distribution of screening tools applied across countries in sub-Saharan Africa.

In total, 45 (38%) of the included studies were from East Africa, 41 (34%) from Southern Africa, 28 (24%) from West Africa, and 5 (4%) from Central Africa. Of the 45 included studies from East Africa, 15 came from Ethiopia (54, 110–123), 9 from Malawi (107, 124–131), 7 from Kenya (18, 27, 132–136), 7 from Tanzania (29, 106, 137–139), 3 from Uganda (102, 140, 141), and 2 each from Rwanda (24, 142) and Mozambique (143, 144). Of the (35) included studies from Southern Africa, 32 were from South Africa (25, 28, 31, 145–172), 7 from Zimbabwe (99, 108, 173–177), and 1 each from Zambia (109) and Eswatini (178). Of the 28 included studies from West Africa, 18 were from Nigeria (16, 97, 98, 100, 101, 103, 179–190), 7 from Ghana (94–96, 191–193), and 3 from Cote d’Ivoire (94–96). The five included studies from Central Africa were two each from Congo (105, 194) and DR Congo (195, 196), and one from Angola (104). Figure 2 summarizes the countries from which the included studies came as well as the tools used in the studies.

Of the 119 studies, 76 (63.9%) included pregnant women only, 53 (36.1%) included postpartum women only, and 10 studies (24, 25, 131, 132, 143, 144, 161, 165, 185, 186) included both pregnant and postpartum women. In two studies (127, 142), the participants were classified as being in the period from pregnancy to 1 year post-delivery. In total, 8 of the 76 studies (104, 128, 145, 152, 153, 161, 175) involved pregnant women living with HIV, while 4 (27, 133, 153, 190) involved adolescent mothers. In five of the studies involving postpartum women, the study participants were living with HIV (109, 135, 154, 161, 174). In one other study (114), the authors included only pregnant women with a history of intimate partner violence.

### Tools available in SSA for screening MMH conditions

**Figure 3:**
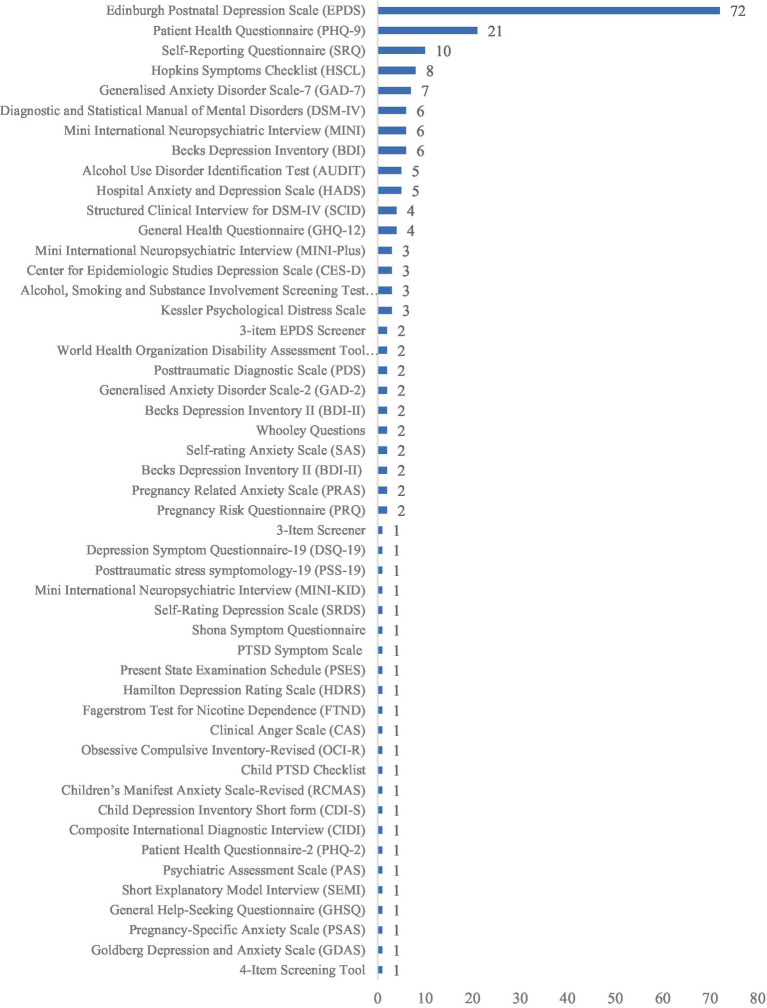
Tools available in countries across Sub-Saharan Africa for screening mental health problems in pregnant and postpartum women in primary care settings.

There were a total of 47 different tools that were either independently or in combination used to screen for seven different MMH conditions (depression, anxiety, suicidal behavior, post-traumatic stress disorder, substance use disorders, obsessive-compulsive disorders, and stress) across the studies (Figure 3). In terms of conditions most frequently screened for, depression was the lead with 68 studies among pregnant and 58 among postpartum women. It was followed by anxiety (21 studies), suicidal behavior (7 studies), post-traumatic stress disorder (6 studies), substance use conditions (5 studies), obsessive-compulsive disorder (OCD) (1 study), and stress (1 study).

Of the 47 different tools described in the studies, the most commonly used tool was the EPDS (see Figure 3). It was used in 72 of the 119 studies (60.5%) to screen for PPD in studies across 13 countries in SSA. Three studies (100, 105, 132) were validation studies, where the performance of EPDS was assessed in postnatal women. In two other studies (125, 126), an abbreviated version of the tool comprising only three items was used to screen depression in postnatal women.

The nine-item Patient Health Questionnaire (PHQ-9) mostly used for screening depression in pregnant and postpartum women was the second most commonly used tool (21 studies) across 10 countries. The questionnaire is designed for individuals to assess their own depression level over the previous 2 weeks. It consists of nine items, and respondents are asked to indicate the frequency of their symptoms using a Likert scale, with options ranging from “0 = not at all” to “3 = nearly every day.” In one study (144), a shortened version of the tool (PHQ-2), comprising only two items (“Little interest or pleasure in doing things”; and “Feeling down, depressed, or hopeless”), was used to screen for depression in pregnant and postpartum women.

The WHO’s Self-Reporting Questionnaire (SRQ) was used to screen for various mental health disorders (such as depression, anxiety, and psychological distress) in 10 studies (102, 107, 125, 126, 129, 130, 146, 164, 188) across four countries in SSA. The tool was mostly translated and adapted to the local context or the respective setting. For example, in two of the included studies (129, 130) conducted in Malawi, the tool was translated into the widely spoken local language—Chichewa.

The Mini International Neuropsychiatric Interview (MINI) was used in 10 studies (31, 102, 121, 126, 143, 144, 149, 153, 160, 172) to screen for various mental disorders including depression, suicidal behavior, and perinatal psychological distress across six countries. In three of the studies (121, 143, 144), the tool was used as the gold standard to evaluate the validity of other screening tools, including the PHQ-9. In one study (153), a version of this tool, specially designed for children and adolescents (MINI-KID) was used to screen for suicidality and self-harm in pregnant adolescents living with HIV in South Africa.

The Generalized Anxiety Disorder (GAD) instrument was used in nine of the included studies (94–96, 160, 168, 190) to screen mainly for anxiety disorders in pregnant and postpartum women. Two variations of this tool—GAD-7 and GAD-2—were used in seven (94–96, 190) and two (160, 168) studies, respectively. The GAD-7 is a 7-item tool, while the GAD-2, a shortened form of the 7-item tool, comprises only two items.

The Hopkins Symptoms Checklist (HSCL), which uses 25 items, was used in eight studies (29, 105, 106, 126, 194, 197, 198) to screen mainly antenatal/postnatal depression and anxiety across three countries. In two studies (124, 125), 15 items instead of the original 25 items of the HSCL were used. The Diagnostic and Statistical Manual of Mental Disorders (DSM-IV) was reported in six studies (108, 140, 171, 177, 190, 195) across five countries, Beck Depression Inventory (BDI) in six studies (112, 113, 118, 133, 146, 148) in three countries, and WHO’s Alcohol Use Disorders Identification Test (AUDIT) in five studies (115, 118, 133, 143, 167) across four countries. The General Health Questionnaire (GHQ) was used in four studies (97, 98, 104, 189) across two countries to screen majorly for depression.

The following tools were used three times each (MINI plus, CES-D, ASSIST, and Kessler Psychological Distress Scale), and two times each (WHODAS, PDS, Whooley Questions, SAS, PRAQ, and PRAS). The rest of the tools (DSQ-19, PSS-19, SRDS, SSQ, PSES, HDRS, FTND, CAS, OCI-R, RCMAS, CDI-S, CIDI, PAS, GHSQ, SEMI, PSAS, GDAS, Child PTSD Checklist, and PTSD Symptom Checklist) were each used once in the studies included in the review.

### Sensitivity and specificity of MH screening tools used in SSA

Sensitivity and specificity are statistical measures commonly used to assess the performance of a tool or test against a gold standard. The sensitivity rate of a screening tool is the proportion of a population such as pregnant women diagnosed as having an MMH condition who truly have that condition. The specificity rate is the proportion of the population diagnosed by the tool as not having the condition who truly do not have that condition. A total of 12 studies reported specificity and sensitivity values for specific screening tools for specific conditions among pregnant or postpartum women. The EPDS, PHQ-9, and the Whooley (for depression) and the SRQ and KPDS (for psychological distress) were the main tools for which reporting of sensitivity and specificity values were done in the papers reviewed. In some cases, the assessment was done for varying screening tool score cut-off points. Generally, if the cut-off point of a test is raised, there are fewer false positives but more false negatives, implying the test is highly specific (measures what it intends to measure to a large extent) but not very sensitive (may miss potential cases). Similarly, if the cut-off point is low, there are fewer false negatives but more false positives indicating the test is highly sensitive but not very specific (Table 1).

The average sensitivity and specificity values for the EPDS were 75.5 and 76.5%, respectively, for four studies (110, 111, 160, 162) at a cut-off point of ≥13; 88 and 74% at a cut-off of ≥12 (175); 88 and 87% at a cut-off point ≥11 for a study (177) and cut-off of 11/12 for a Shona version (108); 78 and 79.5% for two studies at a cut-off point of ≥10 (124, 125); and 87 and 78.5% at a cut-off of ≥8 (99, 133). The PHQ-9 at a cut-off of ≥10 was reported to have a sensitivity of 66% and specificity of 76% (160), while a cut-off of ≥9 gave a 72 and 79% (144) and PHQ-2 at ≥2 gave an average of 74.5 and 70% sensitivity and specificity, respectively, for the two studies (144, 160). The Whooley was reported to have a sensitivity of 75 and 82% and the Whooley +help reported 75 and 82%, respectively, both at a cut-off of 2 by the same study (160).

Psychological distress was measured using the SRQ with a sensitivity of 72% and sensitivity of 96% at a cut-off of ≥10 (99) and 82 and 66%, respectively, at a cut-off of ≥8 (93). The KPDS-10 reported a sensitivity and specificity of 80 and 79% at a cut-off of 11 and KPDS-6 reported 74 and 85% at a cut-off of 8 by the same study (160).

### Potential for adaptation and routine use in SSA health systems

#### Understandability of the tools

Understandability of the tools by those being administered was reported in a total of 15 (96, 108, 115, 121, 124, 127–129, 131, 143, 144, 157, 167, 171, 180) of the 119 studies. Five studies reported difficulty in understanding the EPDS (127, 157), the PHQ-9 (128), and the GAD-7 (96), which was attributed to the low literacy level and construct of the questions due to the culture and context of the study population requiring rewording of the questions to make them relevant.

#### Ease of use

In total, 12 studies stated they found the tools HAD (97), EPDS (100, 103, 129, 131, 157, 162, 168), AUDIT (133, 143), SRQ (107, 129), and the Whooley (160) easy to use especially with reference to busy primary healthcare settings. A few researchers modified the tools (107, 168), used shorter versions of the original tools (162), or modified the scoring (129).

### Adaptability

Related to how the screening tools suspect MMH conditions in SSA, 57 studies identified that the reported adaptation of the tools was predominantly translated into local languages based on the population of interest. Thirty-two studies (56%) involved adaptations of the EPDS, with most focusing on translations into local languages. One study, however, explored culturally relevant terminology for depression (108), incorporating local syndromes (61) and adapting the EPDS for self-administration via mobile technology (149). The EPDS was translated into Yoruba (101, 103, 185), Igbo (100, 182), Afrikaans, Zulu, isiXhosa, Swahili (27, 28, 133, 145, 148, 151, 154, 157, 159, 160, 162, 170), Chichewa (124, 125, 129, 130, 167), and Shona (108, 176). The PHQ-9 was also adapted (94–96, 121, 144, 160, 186) through translation into local languages for administration.

### Type of health staff administering the screening tool

In total, 69 out of 119 studies (52%) reported on personnel who administered the questionnaires (screening tools) in their studies. The cadre of health staff ranged from medical doctors and students (100, 102, 156, 166, 177, 193, 197); nurses, midwives, and nursing students (17, 18, 111, 113, 117, 120–122, 127, 128, 141, 148, 151, 163, 164, 166); clinical psychologist or with a background in psychology (27, 160, 166); (community) health staff, mental health officer; background in mental health or social worker (99, 132, 135, 149, 168, 172, 196); participant (self) administration (28, 148, 180); and non-health staff (108, 130). The rest were categorized as research assistants without specification of their cadre and whether or not they were from the health sector.

## Discussion

This systematic review set out to identify screening tools currently available for detecting MMH conditions in the SSAn health systems, what is known about their sensitivity and specificity, and their potential usefulness for use at scale to screen for MMH conditions in primary care settings in Sub-Saharan African health system context. As such, it addresses an important gap in the published literature about the implementation and appropriateness of tools within the context of SSA which are developed elsewhere (59–63).

In relation to what screening tools are currently available, there is clearly a large number with 47 (19) mental health screening tools that have been used either independently or in combination to screen for various MMH conditions across countries in SSA. Tools for screening for depression, especially for PPD dominate with the EPDS, developed from a combination of several screening tools – Irritability, Depression and Anxiety Scale (IDA), the Hospital Anxiety and Depression Scale (HAD), and the State of Anxiety and Depression scale (SAD) (50, 199), being the most common tool used for assessing MMH conditions. It was used to assess for depression in pregnant as well as postpartum women. Although the tool was mainly developed for the assessment of PPD, it was found to be useful for assessing depression in pregnant women.

Why is this tool so commonly preferred, given there are multiple other screening tools available for depression as well as for other mental health conditions with approximately 37 least commonly (scales with a frequency of 3 and below) screening tools (e.g., CES-D, PRAS, and HDRS) for assessing MMH conditions? Moreover, why is the predominant focus on maternal depression, even though there are other MMH conditions, such as bipolar affective disorder that also affect pregnant and postpartum women? Why do the findings of this review suggest low prioritization of other mental health conditions in the pregnancy and postpartum period? A possible explanation may be as Cox and Holden (50) indicate that the experiences of clinicians in both developed and developing countries reveal a high incidence of depression among women during the pregnancy and postpartum period causing distress due to the effect on their social interactions and that of the infant. The physiological changes occurring during the peripartum period, the cultural norms surrounding the period, and how this defines what is an acceptable expression of emotions or behavior by the woman may make women more prone to depressive mental health disorders as compared to other kinds of mental health challenges. It may also be that the under-researched nature of MMH is itself limiting the research questions that are being asked in this area and the answers sought. It will be important to conduct a review of the prevalence of MMH conditions and relate that information to what kind of mental health conditions should be screened for in pregnant and postpartum women. Given the variety of tools available and the variety of conditions that can be screened for, it is important to be sure if the predominant focus on postpartum and other kinds of depression in maternal health clients should remain or whether it is time for advocacy to screen more generally for other potential mental health problems.

Beyond what a screening tool is designed to detect, its specificity and sensitivity are also important. Since specificity and sensitivity tend to be inversely related it is not possible to have one perfect test. Generally, it is better that a first-line screening test is highly sensitive so that as many potential cases are identified as possible. The cases identified at screening can then be narrowed down by using tools with higher specificity. The specificity and sensitivity of specific screening tools for MMH were determined by studies that sought to establish the psychometric properties of the tools within the population of interest to validate the tools. For instance, the sensitivity of the EPDS was found to be 86%, signifying the proportion of depressed women who truly had depression and with a specificity of 78% indicating the proportion of women who were not depressed who were truly not depressed at a cut of 12/13 score. It was noted, however, that these figures vary in the studies reviewed because of different factors. First, the cut-off points used by researchers in their studies were not standardized which could have affected the sensitivity and specificity of the tools in identifying the condition of interest. Additionally, it was also deduced that the SSA population has a different socio-demographic characteristic from the population where the tools were developed; therefore, some researchers translated the tools into local languages or revised them to make them culturally acceptable. This is in line with findings from earlier reviews (59, 60) both in Africa and non-African LMIC (200, 201) on screening tools for assessing depression and anxiety among pregnant and postpartum women. These adaptations could have been a contributory factor that affected the outcome of the specificity and sensitivity of the tool in capturing the condition of interest. Findings from this review suggest that some studies did not validate screening tools used within the population and condition of interest because sometimes the researchers use sensitivity and specificity values from the psychometric properties of the tool or rely on values documented from studies among a similar population from another country. This may possibly be because of resource constraints (time and financial) and or lack of skill on the part of the research team. The tools developed elsewhere may be generally appropriate to SSA; however, some components of each sensitivity and specificity values are probably due to the differences in contexts in which the tools were developed and applied.

The EPDS screening tool was the most acceptable (based on versatility and sensitivity) for screening for depression among postpartum women. The tool has been used to screen for depression both in the antenatal and postnatal periods and has been found to be valid in identifying women who may be depressed. However, Murray and Cox (202) proposed a higher cut-off point of 14/15 during the antenatal period after validating the EPDS among a sample of 100 women 28 and 34 weeks of pregnancy living in the United Kingdom. Contrary to this proposition, none of the studies reporting on the performance of the EPDS among pregnant women used this suggested cut-off point. Additionally, the set of questions does not specifically cite the postpartum period making it easy to be used in multiple situations even outside pregnancy and the postpartum period (50). Despite some contextual issues raised about the EPDS by some researchers on African and non-African populations (60, 108, 200, 201, 203, 204), from this systematic review, it appears to be the tool with evidence for acceptability, adaptability, ease of use, and effectiveness in identifying potential women who may be depressed during and after pregnancy. It can only identify depression which requires the use of other tools to complement screening for other relevant mental health conditions such as bipolar disorder and anxiety. This may adversely affect the duration of administration, leading to patient fatigue and worker overload for the primary healthcare staff.

Contextual issues related to screening tools developed in the Global North and used in LMIC (205–207) and other non-native English-speaking populations even when emigrated to English-speaking HICs have been well documented (200, 201, 204, 208). Considering that cultural nuances and language greatly affect the expression of distress and symptoms of psychiatric conditions, translations of screening tools do not adequately capture the assessment of the construct and cross-cultural reliability of these tools (207). Our review of the publications reporting on the screening tools for MMH conditions predominantly translated these tools developed and validated for populations in the HIC into languages specific to the population of interest. However, this practice may not be entirely accurate in capturing the context and nuances of the language and culture of the population. In administering the questionnaires by an interviewer as was reported by most of the papers in this review, the interviewer may have discussions in an attempt to clarify some of the constructs through engagement in their local dialect introducing a potential margin of error. This is similar to the finding from a systematic review of tools for assessing behavioral challenges in LIMCs (206). Indeed, using tools validated decades prior from a population similar to the population of interest may not be ideal, as the population may have changed due to the effects of globalization influencing their culture. These influences may need to be considered in the interpretation of results from such studies as the persistent use of these tools indirectly promotes them as cross-culturally appropriate as argued by other researchers (206).

The answer to our third question related to the potential for using any of the tools identified in primary care settings in SSA is influenced by properties of the tools such as understandability of the tool and ease of use, especially by the cadres of staff who are likely to administer the tools or the clients if they are self-administered, and adaptability to context. Reporting on the properties of specific screening tools used in screening for MMH conditions is not routinely done by researchers. From this systematic review, only 12% reported on the understandability of the questions of the screening tool by research participants, 10% on ease of use by researchers, 48% adaptability, and 52% on a specific cadre of health staff or otherwise who administered the tools with nurses, midwives, and nursing students being the most used staff category. Although these may have been noted during the research process and probably discussed among the research team but not formally documented as part of communicating the research outcome to potential readers of the papers. Not documenting this important information on screening tools for detecting MMH conditions deprives the research community and clinicians of essential guidance in appraising these tools holistically in their usage in research or clinical settings.

There are obviously methodological challenges affecting the outcome of the use of these screening tools (52) as well as the complexity of pregnancy and the postpartum period including the hormonal changes, genetic predisposition to mental health conditions, and the environment further influenced by cultural variations in different populations affecting systems, and structures for support. A key population of women who are most predisposed to mental health conditions specifically those who lose their pregnancy before the age of viability or after viability such as stillbirth or death of the fetus from any cause were significantly missing from the studies included in this review, which reported the use of different screening tools. This may be due to the postnatal period primarily focused on a positive outcome of pregnancy and not the woman regardless of the outcome (live child or otherwise).

An overarching finding from our review is that the EPDS appeared to be the most effective, acceptable, user-friendly, and adaptable screening tool for identifying MMH conditions and depression among pregnant and postpartum women in SSA in primary care settings. An ultra-shorter 5-item and 3-item versions have been proposed to address the challenge of work overload for its usage in routine assessment in primary care. There was, however, a wide variation in methodology in the usage of the tool such as the cut-off points from the original 13 or more, an attempt to diagnose or reclassify depression into severity using different cut-off points as well as documentation of the attributed of the tools in the methodology of the papers.

### Limitation of the study

Although the study systematically reviewed the psychometric properties of published papers reporting on a wide range of tools, a significant limitation is the variability in methodological approaches used in the included studies. This variability includes differences in study designs, sample populations, and the cut-off for tools, particularly in validation studies. These have an impact on the outcome and comparability and generalizability of the findings. Additionally, the scanty documentation on the specific attributes of the tools used, such as understandability, ease of use, and adaptability. This lack of detailed and standardized reporting limits the ability to draw robust conclusions about the effectiveness and applicability of the tools across different contexts.

## Conclusion

The EPDS, PHQ-9, Whooley Questions, SRQ, and KPDS were predominantly reported to be effective in screening for MMH problems, demonstrating good sensitivity and specificity across various validation studies. Several studies highlighted the EPDS, Whooley Questions, AUDIT, and HAD as user-friendly and easy to administer, even in busy primary healthcare settings. Additionally, the EPDS and PHQ-9 were frequently noted for their adaptability, with the EPDS being widely translated into local languages and adapted for mobile technology to facilitate self-administration. Among the tools reviewed, the EPDS emerged as the most commonly used, effective, acceptable, user-friendly, and adaptable screening tool for depression in pregnant and postpartum women in SSA’s primary healthcare settings.

However, mental health conditions during this period beyond depression include other conditions, which may be prevalent during this critical period that are not screened for. There is a need to expand advocacy and research to include other prevalent mental health conditions, and address the associated morbidity, to improve the well-being of women and their children during this important period.

### Recommendations

The findings from this study provide some recommendations for consideration at three key levels: clinical practice, policy, and future research.

#### For practice

Health authorities and primary care providers in SSA should integrate EPDS into routine antenatal and postnatal care. The EPDS has demonstrated high effectiveness, usability, and adaptability, making it an ideal tool for the early detection of MMH distress. Additionally, the short and ultra-short versions of available tools can be incorporated into routine care to reduce the burden on practitioners.

Comprehensive training programs should be developed for healthcare providers on the use of EPDS and other effective screening tools such as PHQ-9, Whooley questions, SRQ, and KPDS. These tools may be incorporated into pre-service curricula for key health staff, including midwives, nurses, and medical students in administering these tools, as this could enhance the accuracy and efficiency of mental health screenings in primary care settings, facilitating better integration into routine practice.

#### For policy

Policymakers should develop and implement clear policies supporting the routine use of validated mental health screening tools in maternal healthcare. These policies should mandate the screening of all pregnant women regardless of the outcome to ensure no woman is left behind in having access to mental health support when they are most vulnerable. Targeted policy and strategy will facilitate the systematic integration of these tools into healthcare protocols, promoting consistent and effective mental healthcare for all pregnant and postpartum women.

#### For future research

Ongoing research and evaluation should be encouraged to continuously assess the effectiveness and acceptability of mental health screening tools in different SSA contexts. The focus should be on expanding research beyond depression to include a range of MMH conditions. Continuous evaluation identifies areas for improvement and adaptation, ensuring that screening tools remain relevant and effective. Expanding research ensures comprehensive MMH care.

Efforts to translate and culturally adapt the EPDS and other effective screening tools into local languages and contexts within SSA should continue. Collaboration between clinicians and researchers is essential in developing and testing these adapted tools. Cultural and linguistic adaptations improve accessibility and acceptability, making the tools more effective in diverse communities. Collaborative efforts ensure the tools are practical and context-appropriate.

The use of MMH screening tools should be standardized among researchers in SSA. Studies conducted outside the original context of the tools should document various field experiences to guide holistic appraisal.

## Data Availability

The original contributions presented in the study are included in the article/Supplementary material, further inquiries can be directed to the corresponding authors.
