# Supplementary material for: Tools for screening maternal mental health conditions in primary care settings in sub-Saharan Africa: systematic review
Source: Front Public Health. 2024 Sep 26;12:1321689. doi: 10.3389/fpubh.2024.1321689 (PMC11466175; doi:10.3389/fpubh.2024.1321689)
Supplement: Supplementary file 1 [file Table_1.docx]

**Table 1: Search Terms**

| **Concept 1** | **Concept 2** | **Concept 3** | **Concept 4** | **Concept 5** |
| --- | --- | --- | --- | --- |
| Screening tools  Diagnostic tools  Screening test  Screening  Screening instrument  Screening technique?  Screening scale  Diagnosis  Diagnostic test  Diagnostic tool  Hopkins symptom checklist  Self-report questionnaire  Center for epidemiological studies depression scale  CESD-S  General health questionnaire  GHQ  Beck depression inventory, BDI (any abbreviation for this?)  Whooley questions The Edinburgh Postnatal Depression Scale EPDS The Patient Health Questionnaire PHQ-9 | Mental health  Mental health problem  Psychological disorder(s)?  Emotional disorder(s)?  Mental disorder(s)  Mental illness  Psychiatric illness  Psychiatric disorder(s)  Depression  Depressive disorder(s)  Anxiety  Bipolar  Bipolar Affective Disorder(s)  Psychosis, Psychotic disorder(s)  Paranoia  Psychopathy, Psychopath  Neurosis, Neurotic disorder(s)  Schizophrenia  Schizophrenic disorder(s)  Suicide, Suicidal?,suicidal ideation  Self-harm  Infanticide  Alcohol dependence  Substance use disorder(s) | Pregnant  pregnancy  Prepartum  Prenatal  Antenatal  Antepartum  During pregnancy  Puerperium  Postpartum  Perinatal  Peripartum  Post-delivery  Maternal  Maternity  Parturient  Parturition  Expectant mother(s)? | Primary healthcare  PHC  Primary care  Polyclinic  Antenatal clinic  Clinic  Hospital  Health centre  Health center  District hospital  Health facility  Outpatient  Outpatient department  OPD  Outpatient clinic  CHPS Compound | Sub-Saharan Africa  SSA  Africa  Sub-Sahara  Angola  Benin  Botswana  Burkina Faso  Burundi  Cameroon  Cape Verde  Central African Republic  Chad  Comoros  Congo  Democratic Republic of Congo  Congo Brazzaville  Cote d'Ivoire  Ivory Coast  Djibouti  Equatorial Guinea  Ethiopia  Gabon  The Gambia  Ghana  Guinea-Conakry  Guinea-Bissau  Kenya  Lesotho  Liberia  Madagascar  Malawi  Mali  Mauritania  Mauritius  Mozambique  Namibia  Niger  Nigeria  Rwanda  Sao Tome and Principe  Senegal  Seychelles  Sierra Leone  Somalia  South Africa  Sudan  Swaziland  Tanzania  Togo  Uganda  Zaire  Zambia  Zimbabwe |
