# Supplementary material for: Tools for screening maternal mental health conditions in primary care settings in sub-Saharan Africa: systematic review
Source: Front Public Health. 2024 Sep 26;12:1321689. doi: 10.3389/fpubh.2024.1321689 (PMC11466175; doi:10.3389/fpubh.2024.1321689)
Supplement: Supplementary file 2 [file Table_2.docx]

**Table 2: Search strategy for PubMed (Adapted to the other databases searched)**

| **Search** | **Query** |
| --- | --- |
| #9 | Search: **(#7) AND (#8)** |
| #8 | Search: **(((((((((((((((((((((((((((((((((((((((((((((((("sub-Saharan Africa") OR (SSA)) OR (Angola)) OR (Benin)) OR (Botswana)) OR ("Burkina Faso")) OR (Burundi)) OR (Cameroon)) OR ("Cape Verde")) OR ("Central African Republic")) OR (Chad)) OR (Comoros)) OR (Congo)) OR ("Cote d'Ivoire")) OR (Djibouti)) OR ("Equatorial Guinea")) OR (Ethiopia)) OR (Gabon)) OR ("The Gambia")) OR (Ghana)) OR (Guinea)) OR ("Guinea-Bissau")) OR (Kenya)) OR (Lesotho)) OR (Liberia)) OR (Madagascar)) OR (Malawi)) OR (Mali)) OR (Mauritania)) OR (Mauritius)) OR (Mozambique)) OR (Namibia)) OR (Niger)) OR (Nigeria)) OR (Rwanda)) OR ("Sao Tome and Principe")) OR (Senegal)) OR (Seychelles)) OR ("Sierra Leone")) OR (Somalia)) OR ("South Africa")) OR (Sudan)) OR (Swaziland)) OR (Tanzania)) OR (Togo)) OR (Uganda)) OR (Zaire)) OR (Zambia)) OR (Zimbabwe)** |
| #7 | Search: **(#5) AND (#6)** |
| #6 | Search: **(((((((((("primary healthcare"[Title/Abstract]) OR (PHC[Title/Abstract])) OR ("universal health coverage"[Title/Abstract])) OR (UHC[Title/Abstract])) OR ("primary care"[Title/Abstract])) OR (polyclinic[Title/Abstract])) OR ("antenatal clinic"[Title/Abstract])) OR (clinic[Title/Abstract])) OR (hospital[Title/Abstract])) OR ("health centre"[Title/Abstract])) OR ("health center"[Title/Abstract])** |
| #5 | Search: **(#3) AND (#4)** |
| #4 | Search: **((((((((((((("pregnant women"[Title/Abstract]) OR (prepartum[Title/Abstract])) OR (peripartum[Title/Abstract])) OR (perinatal[Title/Abstract])) OR (prenatal[Title/Abstract])) OR (antenatal[Title/Abstract])) OR ("during pregnancy"[Title/Abstract])) OR (postpartum[Title/Abstract])) OR (maternal[Title/Abstract])) OR (maternity[Title/Abstract])) OR (parturient[Title/Abstract])) OR (antepartum[Title/Abstract])) OR ("post-delivery"[Title/Abstract])) OR (puerperium[Title/Abstract])** |
| #3 | Search: **(#1) AND (#2)** |
| #2 | Search: **(((((((((((((((((((((((("Mental health"[Title/Abstract]) OR ("Mental health problem"[Title/Abstract])) OR (psychological[Title/Abstract])) OR (emotional[Title/Abstract])) OR ("mental disorder"[Title/Abstract])) OR ("mental illness"[Title/Abstract])) OR ("psychiatric illness"[Title/Abstract])) OR ("psychiatric disorder"[Title/Abstract])) OR (depression[Title/Abstract])) OR ("depressive disorder"[Title/Abstract])) OR (anxiety[Title/Abstract])) OR (bipolar[Title/Abstract])) OR ("bipolar affective disorder"[Title/Abstract])) OR (psychosis[Title/Abstract])) OR (psychoses[Title/Abstract])) OR (paranoia[Title/Abstract])) OR (psychopathy[Title/Abstract])) OR (neurosis[Title/Abstract])) OR (schizophrenia[Title/Abstract])) OR ("schizophrenic disorder"[Title/Abstract])) OR (suicide[Title/Abstract])) OR (self-harm[Title/Abstract])) OR (infanticide[Title/Abstract])) OR ("alcohol dependence"[Title/Abstract])) OR ("substance use disorders"[Title/Abstract])** |
| #1 | Search: **(((((((((((((((((((("screening tools"[Title/Abstract]) OR ("screening instrument"[Title/Abstract])) OR ("screening test"[Title/Abstract])) OR ("screening scale"[Title/Abstract])) OR (screening[Title/Abstract])) OR ("diagnostic tool*"[Title/Abstract])) OR ("diagnostic instrument"[Title/Abstract])) OR (diagnosis[Title/Abstract])) OR ("Hopkins symptom checklist"[Title/Abstract])) OR ("Self-report questionnaire"[Title/Abstract])) OR ("Center for epidemiological studies depression scale"[Title/Abstract])) OR (CESD[Title/Abstract])) OR ("General health questionnaire"[Title/Abstract])) OR (GHQ[Title/Abstract])) OR ("Beck depression inventory"[Title/Abstract])) OR ("Whooley questions "[Title/Abstract])) OR ("Edinburgh Postnatal Depression Scale"[Title/Abstract])) OR (EPDS[Title/Abstract])) OR ("Patient Health Questionnaire"[Title/Abstract])) OR (PHQ[Title/Abstract])) OR (PHQ-9[Title/Abstract])** |
