# Supplementary material for: Tools for screening maternal mental health conditions in primary care settings in sub-Saharan Africa: systematic review
Source: Front Public Health. 2024 Sep 26;12:1321689. doi: 10.3389/fpubh.2024.1321689 (PMC11466175; doi:10.3389/fpubh.2024.1321689)
Supplement: Supplementary file 3 [file Table_3.pdf]

Tools for screening maternal mental health conditions at primary health setting in sub-Saharan Africa

(Study selection flowchart)

Step 1: Does the study focus on a mental health problem in pregnant or postpartum* women?
(See list of MMH conditions)

*Postpartum defined as up to 12 months after delivery

Maternal Mental Health Problems

Depression	Substance use disorder
Psychosis	Suicidal ideation
Dysthymia	Bipolar Affective Disorder
Obsessive-compulsive disorder	Paranoia
Posttraumatic stress disorder	Psychopathy
Anxiety	Neurosis/neurotic disorder
Schizophrenia	Self-harm
Infanticide	Alcohol dependence
Substance use disorder	Anorexia nervosa
Bulimia	

NO → **Reason: Wrong focus**
EXCLUDE
Label:
- not pregnant women
- not postpartum women
- not mental health problem

YES ↓

Step 2: Was the study conducted in a primary healthcare setting?

NO → **Reason: Wrong healthcare setting**
EXCLUDE
Label:
- Specialized healthcare setting
- Tertiary healthcare setting

YES ↓

Step 3: Was the study conducted in a country in sub-Saharan Africa?
(See list of countries in SSA)

SSA countries

Angola	Madagascar
Benin	Malawi
Botswana	Mali
Burkina Faso	Mauritania
Burundi	Mauritius
Cameroon	Mozambique
Cape Verde	Namibia
Central African Republic	Niger
	Nigeria
Chad	Rwanda
Comoros	Sao Tome and Principe
Congo	Senegal
Cote d'Ivoire	Seychelles
Djibouti	Sierra Leone
Equatorial Guinea	Somalia
	South Africa
Ethiopia	Sudan
Gabon	Swaziland
The Gambia	Tanzania
Ghana	Togo
Guinea	Uganda
Guinea-Bissau	Zaire
Kenya	Zambia
Lesotho	Zimbabwe
Liberia	

NO → **Reason: Wrong setting**
EXCLUDE
Label: not SSA

YES ↓

Step 4: Does the study state the tool/technique/test used in screening/diagnosing the mental health condition?

NO → **Reason: undocumented**
EXCLUDE
Label: tool not stated

YES ↓

Step 5: Is this a primary study?
- RCTs, Cohort, case-control, cross-sectional, diagnostic accuracy study, etc.

NO → **Reason Wrong study type**
EXCLUDE
Label:
- Review
- Commentary
- Opinion

Mavbe/unclear for inclusion → **TO BE DECIDED**

TO BE DECIDED — YES → INCLUDE
TO BE DECIDED — NO → EXCLUDE

YES ↓

INCLUDE

EXCLUDE
