# Supplementary material for: Tools for screening maternal mental health conditions in primary care settings in sub-Saharan Africa: systematic review
Source: Front Public Health. 2024 Sep 26;12:1321689. doi: 10.3389/fpubh.2024.1321689 (PMC11466175; doi:10.3389/fpubh.2024.1321689)
Supplement: Supplementary file 4 [file Table_4.docx]

**Table 4: Characteristics of studies included in the systematic review**

|  | **Study ID** | **Country** | **Study aims/objective** | **Study setting*** | **Study participants**** | **Age***** | **MMH condition** | **N** | **Screening tool(s)** |
| --- | --- | --- | --- | --- | --- | --- | --- | --- | --- |
| 1 | Abebe et al 2019 (94) | Ethiopia | To assess prevalence and associated factors of postpartum depression among mothers attending maternal and child health clinics | Primary care (postnatal clinic) | Postpartum women | Mean (24.3)  SD (3.83) | Postpartum depression | 511 | EPDS |
| 2 | Abiodun 2006 (88) | Nigeria | To determine prevalence and associated factors for postnatal depression in a primary healthcare | Primary care | Postpartum women | Mean (27.9)  SD (5.9) | Postnatal depression | 360 | EPDS  PSES |
| 3 | Acheanpong et al 2022 (187) | Ghana | To estimate prevalence and risk factors associated with depression during pregnancy | Primary care (Antenatal clinic) | Pregnant women | Mean (27.9)  SD (6.55) Range (18–47) | Antenatal depression | 1433 | PHQ-9 |
| 4 | Ayele et al 2016 (102) | Ethiopia | To determine prevalence and identify associated factors for antenatal depression | Primary care (Antenatal clinic) | Pregnant women | Median (25) Range (28–22) | Antenatal depression | 388 | BDI |
| 5 | Bakare et al 2017 (171) | Nigeria | To improve access of mothers screened positive for postpartum depression to interventions and by that resolve their symptoms of depression, improve growth outcomes in their infants and promote mother-child bonding | Primary care (Immunization clinics) | Postpartum women | Mean (29.0)  SD (3.8) Range (22-35) | Postpartum depression | 3903 | EPDS |
| 6 | Barthel et al 2015-study 1 (78) | Côte d'Ivoire | To explore psychometric properties of the 9-item Patient Health Questionnaire (PHQ-9) in pregnant women | Primary care | Pregnant women  (third trimester) | Mean (28.2) SD (5.8) | 1. Antenatal depression 2. Postpartum depression 3. Anxiety | 639 | PHQ-9 GAD-7 |
| 7 | Barthel et al 2015-study 2 (78) | Ghana | To explore psychometric properties of the 9-item Patient Health Questionnaire (PHQ-9) in pregnant women | Primary care (Komfo Anokye Teaching Hospital) | Pregnant women (third trimester) | Mean (29.5) SD (4.88) | 1. Antepartum depression 2. Postpartum depression 3. Anxiety | 389 | PHQ-9 GAD-7 |
| 8 | Barthel et al 2016-study 1 (79) | Côte d'Ivoire | To examine trajectories of ante- and postpartum generalized anxiety symptoms in West-African women and their associations with mother and child characteristics | Primary care | Pregnant women in (third trimester) | Mean (28.9) SD (5.5) Range (18–46) | 1. Antepartum depression 2. Ante- and postpartum anxiety | 778 | PHQ-9 GAD-7 |
| 9 | Barthel et al 2016-study 2 (79) | Ghana | To examine trajectories of ante- and postpartum generalized anxiety symptoms in West-African women and their associations with mother and child characteristics | Primary care (Komfo Anokye Teaching Hospital) | Pregnant women  (third trimester) | Mean (28.9) SD (5.5) Range (18–46) | 1. Antepartum depression 2. Ante- and postpartum anxiety | 778 | PHQ-9 GAD-7 |
| 10 | Bernatsky et al 2007  (89) | Angola | To assess the mental health status of pregnant women living with HIV compared with other groups of pregnant women | Primary care (Rural Health Post) | 1. Pregnant women (HIV +ive/-ive) | Mean (23.8) HIV positive Mean (22.9) without HIV | *Poor mental health *HIV | 23 (HIV-positive)  134 (without HIV) | GHQ-12 |
| 11 | Brittain et al 2017 (149) | South Africa | To explore HIV-related stigma and antenatal depressive symptoms | Primary Care (Antenatal Clinic) | HIV-positive pregnant women initiating ART | Median (28) Inter-quartile range (24–32] | Antenatal depression | 623 | EPDS |
| 12 | Chibanda et al 2010 (93) | Zimbabwe | To assess the validity if the Shona version of the EPDS in a cohort of postpartum women attending urban primary care clinics in Zimbabwe compared with the DSM-IV criteria for major depression as the gold standard | Primary care (postnatal clinic) | Postpartum depression | Mean (25) | 114 | 210 | EPDS  DSM-IV |
| 13 | Cyimana et al 2010 (85) | Zambia | To study the contribution of HIV/AIDS to postnatal depression among women receiving postnatal care | Primary care (University Teaching Hospital) | Postpartum woman presenting to the hospital with known HIV status | Range (30-34 and ≥35) | Postnatal depression | 229 | EPDS |
| 14 | Dlamini et al 2019 (162) | Swaziland | To describe the prevalence and factors associated with postpartum depression among women seeking postnatal and child welfare services at a Primary Healthcare Facility | Primary care | Postpartum women | Range (18-45) | Postpartum depression | 114 | EPDS |
| 15 | Guo et al 2014-study 1 (80) | Côte d’Ivoire | To explore maternal Parenting Stress levels and influence of ante- and postnatal depression and anxiety on Parenting Stress among low-risk pregnancies | Primary care  (Community Hospital) | Pregnant women  (third trimester) | Mean (29.1) SD (5.5) | 1. Perinatal Depression 2. Perinatal Anxiety | 577 | PHQ-9 GAD-7 |
| 16 | Guo et al 2014-study 2 (80) | Ghana | To explore maternal Parenting Stress levels and influence of ante- and postnatal depression and anxiety on Parenting Stress among low-risk pregnancies | Primary care (Komfo Anokye Teaching Hospital) | Pregnant women  (third trimester) | Mean (29.1) SD (5.5) | 1. Perinatal Depression 2. Perinatal Anxiety | 577 | PHQ-9  GAD-7 |
| 17 | Harrington et al 2018 (112) | Malawi | To estimate the association of probable antenatal depression with postpartum HIV among pregnant women | Primary care | Pregnant women living with HIV | Median (26) Range (22–30) | Probable antenatal depression | 299 | EPDS |
| 18 | Kakyo et al 2012 (125) | Uganda | To explore the factors associated with postpartum depressive symptoms | Primary care | Postpartum women | Mean (24)  SD (4.33) | Postpartum depression | 202 | EPDS |
| 19 | Kugbey et al 2021 (176) | Ghana | To examine prevalence and correlates of prenatal depression, anxiety and current suicidal behaviors among pregnant women | Primary care (Antenatal clinics in a teaching and Municipal hospital) | Pregnant women | Range (26-35) | 1. Prenatal Depression 2. Anxiety 3. Suicidal behaviours | 214 | HADS |
| 20 | MacGinty et al 2020 (151) | South Africa | To identify antenatal maternal psychological distress risk factors and determine whether antenatal maternal psychological distress was associated with infant birth and developmental outcomes in South Africa | Primary care | Pregnant women | Mean (26)  SD (5.70) | 1. Antenatal psychological distress 2. Antenatal depression 3. PTSD 4. Alcohol use disorder | 961 | SRQ-20 BDI  ASSIST  Modified PTSD symptom scale |
| 21 | Mahenge et al 2013 (181) | Tanzania | To determine the prevalence of intimate partner violence against women during pregnancy and its associated mental health symptoms | Primary care (National Hospital Antenatal Clinic) | Pregnant women | Mean (29) | 1. Anxiety 2. Depressive symptoms 3. PTSD | 1180 | HSCL-25  PDS |
| 22 | Mahenge et al 2015 (182) | Tanzania | To establish the prevalence of symptoms of anxiety, depression, and post-traumatic stress disorder (PTSD) among women attending prenatal care in Tanzania, and identify associated factors. | Primary care (National Hospital Antenatal Clinic) | Pregnant women | Mean (29)  Range (17–43) | 1. Anxiety 2. Depression 3. PTSD | 1180 | HSCL  PDS |
| 23 | Manikkam and Burns 2012 (16) | South Africa | To determine the prevalence and risk factors associated with antenatal depressive symptoms in a KwaZulu-Natal population | Primary care (Antenatal Clinic) | Pregnant women attending ANC | Mean (27)  SD (6.1) Range (14-46) | Antenatal depression | 387 | EPDS |
| 24 | Mokwena and Masike 2020 (152) | South Africa | To determine the prevalence and factors associated with postnatal depression | Primary care | Postpartum women | Mean (28)  SD (5.91) Range (18-45) | Postnatal depression | 406 | EPDS |
| 25 | Necho et al 2020 (95) | Ethiopia | To determine the magnitude of postpartum depression and its correlates | Primary care | Postpartum women | Mean (29.9) SD (6.39) | Postpartum Depression | 378 | EPDS |
| 26 | Nhiwatiwa et al 1998 (83) | Zimbabwe | To determine whether a brief psychiatric screening questionnaire used in the 8th month of pregnancy can predict postnatal mental disorder | 1. Primary care clinic 2. Traditional birth attendants | Pregnant women | Mean (25.3) SD (6.8)  Mean (23.5) SD 5.7) | Postnatal mental Disorders | 500 | SSQ |
| 27 | Odinka et al 2018 (172) | Nigeria | To assess the prevalence of postpartum depression and anxiety in relationship to marital satisfaction in low-risk women | Primary care | Postpartum women | Mean (29.7) SD (4.87) Range (20–46) | 1. Postpartum depression 2. Postpartum anxiety | 309 | HADS |
| 28 | Stellenberg et l 2015 (153) | South Africa | To determine the prevalence of postnatal depression (PND) and risk factors associated with PND | Primary care (Rural Community Clinic) | Postpartum women | Not reported | Postnatal depression | 159 | EPDS BDI |
| 29 | Stewart et al 2009 (92) | Malawi | To validate the Chichewa version of the Self-Reporting Questionnaire (SRQ) as a brief screening measure for maternal depression | Primary care  (Rural Child Health Clinic) | Postpartum women | Mean (24.8) SD (5.8) | Maternal depression | 114 | SRQ |
| 30 | Stewart et al 2014 (114) | Malawi | To validate the multi-dimensional scale of perceived social support (MSPSS) and the relationship between social support, intimate partner violence and antenatal depression | Primary care  (Antenatal Clinic) | Pregnant women | Mean (25.1)  SD (6.22) | Antenatal depression | 583 | EPDS  SRQ |
| 31 | Umuziga et al 2015 (126) | Rwanda | To determine Common Perinatal Mental Disorders in a district hospital | Primary care  (District hospital) | Postpartum women | Range (25-29) | 1. Perinatal depression 2. Perinatal anxiety | 165 | EPDS  SAS |
| 32 | Uwakwe 2003 (84) | Nigeria | To determine the rate of depression in a group of postpartum Nigerian women and to validate the Edinburgh Postnatal Depression Scale (EPDS) in this group | Primary care (postnatal clinic) | Postpartum women | Mean (28.9) SD (4.2) Median (29) Range (18–39) | Depression | 225 | EPDS  SRDS |
| 33 | Heyningen et al 2017 (154) | South Africa | To investigate the prevalence and predictors of antenatal anxiety disorders among low-income women living with psychosocial adversity | Primary care (Community clinic) | Pregnant women | Mean (27)  SD (5.8) | Common Perinatal Mental Disorders (CPMD) | 376 | MINI Plus |
| 34 | Wubetu et al 2020 (98) | Ethiopia | To determine the prevalence of postpartum depression and factors associated with postpartum depression in postnatal care attendees | Primary care (health centres and antenatal clinic in a referral hospital) | Postpartum women | Range (25-45) | Postpartum depression | 308 | EPDS |
| 35 | January & Chimbari 2018 (157) | Zimbabwe | To explore the prevalence and associated factors of postnatal depression among women attending postnatal care services in two rural districts | Primary care  (Rural health centres) | Postpartum women | Mean (23.7)  SD (6.14) | Postnatal Depression | 453 | EPDS  PHQ-9  CES-D |
| 36 | Kimbui et al 2018 (117) | Kenya | To assess the prevalence of depression, substance abuse and associated risk factors | Primary care  (Community Health Centre) | Pregnant adolescents | Mean (17.3)  SD (1.9)  Range (14-18) | Depression  Substance misuse | 212 | EPDS  AUDIT BDI II |
| 37 | Mandell et al 2021 (155) | South Africa | To determine BP, depression and suicidal ideation among pregnant women with HIV | Primary care  (Community Health Centre) | Pregnant women | Mean (28.5) SD (5.8) | Depression  Suicidal ideation | 217 | EPDS-10 |
| 38 | Mebrahtu et al 2018 (158) | Zimbabwe | To assess the association between Postpartum mental health and cognitive development of HIV exposed infants in Zimbabwe | Primary care (Rural clinics) | Postpartum women with HIV | Mean (31.5) SD (6.3) | 1. Depression 2. Stress | 397 | EPDS PSI-SF |
| 39 | Nyamukoho et al 2019 (159) | Zimbabwe | To determine factors associated with depression in HIV pregnant women | Primary care  (Antenatal clinics) | Pregnant women with HIV | Mean (26.6) SD (4.5) | Depression | 197 | EPDS |
| 40 | Nydoo et al 2017 (156) | South Africa | To compare depression scores between newly diagnosed HIV-infected and HIV uninfected pregnant women | Primary care (Antenatal clinics) | Pregnant women | Mean (25.0) SD (5.3) | Depression | 102 | EPDS |
| 41 | Ongeri et al 2018 (5) | Kenya | To estimate the prevalence and incidence of significant postpartum depressive symptoms in pregnant women | Primary care (Outpatient Maternal and Child Health Clinic) | Pregnant women | Median (25.5) IQR (22.8-28) | Depression | 188 | EPDS |
| 42 | Stewart et al 2013 (113) | Malawi | To compare the Edinburg Postnatal Depression Scale (EPDS and Self Reporting Questionnaire (SRQ) for the identification of antenatal depression | Primary care (Antenatal clinic) | Pregnant women | Median (24.0) IQR (20-29) | Depression | 224 | EPDS  SRQ  SCID |
| 43 | Tungchama et al 2016 (173) | Nigeria | To determine the role of psychosocial factors in postpartum depression | Primary care Postnatal unit of the Department of Obstetrics and Gynaecology and Child Welfare Clinic | Postpartum women | Mean (27.0) SD (6.0) | Depression | 531 | EPDS  SCID |
| 44 | Wong et al 2017 (129) | South Africa | To compare depression, alcohol use and stigma in younger versus older HIV-infected pregnant women initiating antiretroviral therapy | Primary care (Antenatal clinic) | Pregnant women with HIV | Mean (22.0) | Depression | 625 | EPDS |
| 45 | Zelalem et al 2020 (99) | Ethiopia | To assess prevalence and correlates of antenatal depression among postpartum women | Primary care (Community Clinic) | Postpartum women | Mean (28.7)  SD (5.23) | Depression | 526 | EPDS |
| 46 | Yamamoto et al 2019 (123) | Tanzania | To explore the potential associations between maternal stress, anxiety and other variables in relation to the practice of geophagy in early pregnancy | Primary care (District hospital and regional referral hospital) | Pregnant women | Mean (26) | 1. Stress 2. Anxiety | 219 | PRAS-revised PSS |
| 47 | Woldetensay et al 2018 (97) | Ethiopia | To validate the 9-item Patient Health Questionnaire (PHQ-9) as a screening instrument for depression among pregnant women | Primary care  (Commonly Health Posts) | Pregnant women | Mean (24.3) SD (5.6) | Depression | 246 | PHQ-9  MINI-plus |
| 48 | Ukaegbe et al 2012 (174) | Nigeria | To assess the prevalence and associated sociodemographic variables of postpartum depression among women of Igbo ethnic origin | Primary care  (Urban Mission Hospital) | Postpartum women | Mean (29.6) SD (4.4) | Depression | 186 | EPDS |
| 49 | Roberts et al 2022 (130) | South Africa | To determine the prevalence of likely common mental disorder among adolescents who have experienced motherhood and are living with HIV | Primary care | Pregnant adolescents with HIV | Median (15) | 1. Depression 2. PTSD 3. Suicidal ideation 4. Anxiety | 1059 | CDI-S MINI-KID RCMAS PSS-19 |
| 50 | Peltzer 2011 (131) | South Africa | To determine the prevalence of depressed mood and associated factors in postnatal HIV-positive women in primary care facilities | Primary care | Postpartum women with HIV | Mean (28.5)  SD (5.8) | Depression | 607 | EPDS |
| 51 | Malemela & Mashegoane 2019 (132) | South Africa | To establish rates of obsessive compulsive disorder amongst   pregnant black African women attending community clinics and to determine the relationship between OCD and prenatal depression, pregnancy-related anxiety and anger during pregnancy | Primary care | Pregnant women | Mean (27.7) SD (6.0) | 1. Depression 2. Anxiety 3. Anger during pregnancy 4. OCD | 206 | EPDS OCI-R PRAS CAS |
| 52 | Bass et al 2008 (90) | Congo | To determine if a post-partum depression syndrome exists among mothers in Kinshasa,  Democratic Republic of Congo, by adapting and validating standard screening instruments | Primary care  (Peri-urban community maternity clinics) | Postpartum women | Not reported | Depression | 213 | EPDS  HSCL |
| 53 | Adewuya et al 2005 (86) | Nigeria | To examine sociodemographic and obstetric risk factors for postnatal depressive symptoms | Primary care (Postnatal and infant clinics) | Postpartum women | Mean (28.37 SD (12.1) | Depression | 928 | EPDS |
| 54 | Aderigbedbe & Gureje 1992 (82) | Nigeria | To validate the GHQ-28 | Primary care (Antenatal clinic of the University College Hospital) | Pregnant women | Mean (28.8) SD (4.6) | 1. Depression 2. Anxiety 3. Phobia | 499 | PAS GHQ |
| 55 | Abrahams et al 2018 (19) | South Africa | To assess factors associated   with food insecurity and depression in a sample of pregnant women | Primary care (Midwife Obstetric Unit) | Pregnant women | Range (18-35) | 1. Depression 2. Suicidality 3. Generalized anxiety disorder 4. Drug dependence 5. Substance dependence | 376 | PSSS  MINI-plus |
| 56 | Kaaya et al 2008 (91) | Tanzania | To detect depressive disorder using a 19-item local instrument in Tanzania | Primary Healthcare Antenatal Clinics | Pregnant women | Mean (24.9); SD (5.7) | 1. Maternal depression | 787 | HSCL-25 DSQ-19 |
| 57 | Emerson et al 2020 (178) | Congo | To investigate association between mental health symptoms and diet and nutritional status of mothers of young children | Primary care | Postpartum women | 29.6 | 1. Anxiety 2. Depression 3. PTSD | 828 | HSCL-25 HTQ |
| 58 | January et al 2015 (160) | Zimbabwe | To determine the prevalence of postnatal depression and establish factors associated with postnatal depression | Primary care (Polyclinic) | Postpartum women | Mean (25.4)  SD (5.6) | Depression | 295 | EPDS |
| 59 | E-Andjafono et al 2020 (179) | DRC | To determine the relationship between mothers' feelings about pregnancy and childbirth, infant´s behaviour and development | Primary Care | Postpartum women | Mean (28) | Maternal depression | 120 | EPDS GDAS) DSM-IV (MINI) Criteria |
| 60 | Dadi et al 2020 (105) | Ethiopia | To explore the possible pathways leading to postnatal depression | Primary care Community hospital and health centres) | Pregnant women | Mean (26.5)  SD (0.2) | 1. Depression 2. Social support | 916 | EPDS |
| 61 | Chorwe & Chipps 2018 (109) | Malawi | To assess the performance of a range of screening instruments in detecting depressive symptoms in antenatal clinics | Primary care (Antenatal Clinics) | Pregnant women | Mean (25.2); SD (5.5) | 1. Depression 2. Social support | 480 | EPDS  HSCL-15  SRQ  PRQ  3-item EPDS screener |
| 62 | Abiodun 1994 (81) | Nigeria | To assess the utility of the Hospital Anxiety and Depression Scale as a screening instrument for anxiety and depressive disorders in non-psychiatric units and a community sample in Nigeria. | Mixture of primary care and in-patients) | Pregnant women | NR | 1. Depression 2. Anxiety |  | GHQ-21 HADS SRQ-20 |
| 63 | Phukuta & Omole 2020 (133) | South Africa | To determine the prevalence and factors associated with PND in the postnatal clinic of a large community health centre | Primary care (Community Health Centre) | Postpartum women | Mean (27) | Depression | 227 | EPDS |
| 64 | Mossie et al 2017 (107) | Ethiopia | To assess antenatal depressive symptoms and associated factors among women | Primary care | Pregnant women | Range (16-30) | Antenatal depression | 209 | BDI |
| 65 | Okeke 2021 (163) | Nigeria | To improve birth outcomes  To study the effect of interventions aimed at improving birth-related outcomes on mental health and depression of pregnant women. | Primary care (180 facilities) | Pregnant women | Mean (24.9) SD (5.9) treatment group  Mean (24.7) SD (5.8) control group | Depression | 8578 | EPDS |
| 66 | Mahenge et al 2018 (121) | Tanzania | To determine the prevalence of physical and/or sexual Adverse Childhood Experiences (ACEs) and IPV and their association to symptoms of postpartum depression | Primary care | Postpartum women | Mean (27) Range (18-48) | Depression | 500 | PHQ-9 |
| 67 | Green et al 2018 (116) | Kenya | To validate the EPDS and PHQ-9 in rural Kenya, while at the same time developing and validating a new instrument that blends items from existing screening tools with local idioms of distress | Primary care | 1. Pregnant women 2. Postpartum women | Mean (28.2) SD (3.4) | Depression | 210 | EPDS PHQ-9 SCID-5-RV |
| 68 | Abrahams et al 2019 (134) | South Africa | 1. To use a cognitive interviewing technique to validate the content and structure of a 4-item screening tool. 2. To adapt the tool accordingly 3. To use receiver operating curve (ROC) analysis to determine the optimum cut-point for identifying pregnant women with symptoms of Common Mental Health Disorders | Primary care | Pregnant women | Mean (27.5) SD (5.7) Mean (15-38) | 1. Depression 2. Anxiety 3. Suicidality | 66 | EPDS 4-item Screening tool |
| 69 | Acheampong et al 2021 (175) | Ghana | To compare the risk of developing adverse maternal and perinatal outcomes between pregnant women with moderate-severe depression and those who had mild depression | Primary care | Pregnant women | Mean (27.5) SD (6.5) | Depression | 360 | PHQ-9 |
| 70 | Adeyemo et al 2020 (164) | Nigeria | To determine the prevalence, risk factors and predictors of PPD among postnatal women, six weeks after delivery | Primary care | Postpartum women | Mean (29.5) SD (5.70) | Depression | 250 | EPDS |
| 71 | Agbaje et al 2019 (3) | Nigeria | To assess the prevalence of depression, anxiety and associated factors in postnatal women | Primary care | Postpartum Women | Range (15-29) | Depression Anxiety | 270 | EPDS HADS-A |
| 72 | Alenko et al 2020 (101) | Ethiopia | To identify sociodemographic and obstetric determinants of antenatal depression among women attending antenatal clinic | Primary care (Antenatal Clinic) | Pregnant women | Mean (25)  SD (4.63) | 1. Depression 2. Substance Use Disorders | 246 | BDI-II ASSIST |
| 73 | Ayele et al 2021 (103) | Ethiopia | To investigate the prevalence and associated factors of antenatal depression among pregnant mothers who had intimate partner violence during pregnancy | Primary care (Antenatal Clinic) | Pregnant women | Range (25-34) | Depression | 409 | EPDS |
| 74 | Brittain et al 2015 (135) | South Africa | 1 To examine the demographic and psychosocial predictors of antenatal depression in pregnant women  2 To explore the associations between antenatal depression and infant birth outcomes | Primary care | Pregnant women | Median (25.8) | Depression | 726 | BDI-II |
| 75 | Brittain et al 2019 (136) | South Africa | To determine the impact of HIV-status disclosure on depression during pregnancy and postpartum among women who tested HIV-positive during the pregnancy | Primary care (Antenatal Clinic) | Pregnant women with HIV | Median (26.9) | Depression | 350 | EPDS |
| 76 | Chorwe-Sungani & Chipps 2018b (109) | Malawi | To assess the validity of a range of instruments for screening depression and to test the utility of combining these instruments for use in antenatal clinics | Primary care (Antenatal clinics) | Pregnant women | Mean (26)  SD (5.7) | Depression | 97 | EPDS 3-item EPDS screener  HSCL-15 SRQ |
| 77 | Fantahun et al 2018 (106) | Ethiopia | To assess the prevalence and associated factors of postpartum depression among mothers attending public health centers | Primary care | Postpartum women | Mean (28.1)  SD (5.0) Median (28) | Depression | 618 | EPDS |
| 78 | Gureje et al 2019 (166) | Nigeria | To compare high-intensity treatment with low-intensity treatment for perinatal depression | Primary Care  (Maternal clinics) | 1. Pregnant women 2. Postpartum women | Mean (24.7) SD (5.7) | Depression | 686 | EPDS |
| 79 | Gureje et al 2019b (165) | Nigeria | To compare a stepped-care package for depression with usual care enhanced by the use of the WHO Mental Health Gap Action Programme intervention guide | Primary maternal clinics | 1. Pregnant  2. Postpartum women | Mean (50.2)  SD (15.0) intervention Mean (44.0) SD (14.5) for the control | Depression | 1178 | PHQ-9 |
| 80 | Heyningen et al 2018 (137) | South Africa | 1. To determine prevalence rates of antenatal depression in South Africa by providing accurate diagnostic data using a structured clinical interview of pregnant women | Primary care | Pregnant women | Range (18-24) | Depression | 376 | MINI-plus |
| 81 | Ikeako et al 2018 (167) | Nigeria | To determine the prevalence and correlates of postpartum depression among women visiting postnatal clinic in a tertiary institution | Primary care  (both primary and secondary obstetric care) | Postpartum women | Mean (26) Range (18-40) | Depression | 132 | EPDS |
| 82 | Nakku et al 2006 (87) | Uganda | 1 To determine the prevalence and associated factors of major depression | Primary care | Postpartum Women | Mean (23.4) SD (4.76) | Depression | 544 | SRQ-25  MINI |
| 83 | Osok et al 2018 (15) | Kenya | To determine the prevalence of depression in pregnant adolescents living in a resource-poor urban settlement of Nairobi | Primary care | Pregnant adolescents | Mean (18) | Depression | 176 | EPDS PHQ-9 |
| 84 | Peltzer et al 2018 (138) | South Africa | To determine prevalence of prenatal and postnatal depressive symptoms and associated factors in perinatal HIV-infected women in community health care centres | Primary care (Community centres) | 1. Pregnant women with HIV 2. Postpartum women with HIV | Mean (28.3) SD (5.7) | Depression | 681 | EPDS |
| 85 | Rochat et al 2013 (139) | South Africa | To test the hypothesis that shortened versions of the EPDS are as effective as longer versions in identifying antenatal depression as determined by a clinical interview diagnostic method | Primary Care | Pregnant women | Mean (24)  SD (4.33) | Depression | 112 | EPDS SCID |
| 86 | Sorsdahl et al 2015 (140) | South Africa | 1 To describe the feasibility of integrating SBIRT among women presenting to antenatal care at a Midwife Obstetric Clinic | Primary care | Pregnant women | Mean (26) Range (16-46) | Depression | 1468 | EPDS  FTAD |
| 87 | Spedding et al 2020 (141) | South Africa | To investigate participants' preliminary responses to a task sharing Problem Solving Technique intervention, and evaluate the feasibility and acceptability of the intervention | Primary care | Pregnant women | ≥25 (71.1%) | 1. Depression 2. Psychological Distress | 38 | EPDS SRQ  ASSIST PSS |
| 88 | Tesfaye & Agenagnew et al 2021 (96) | Ethiopia | To assess the prevalence of antenatal depression and associated factors among women attending antenatal care. | Primary care | Pregnant women | Range (25-29) | Depression | 314 | PHQ-9 |
| 89 | Tuthill et al 2017 (142) | South Africa | To identify levels of perinatal depression on infant feeding outcomes | Primary care | 1. Pregnant women  2. Postpartum women | Mean (27.6) SD (6.08) | Depression | 68 | PHQ-9 |
| 90 | Umuziga et al 2020 (12) | Rwanda | To determine the prevalence and associated factors of Common Perinatal Mental Disorders (anxiety and depression) | Primary care | 1. Pregnant women  2. Postpartum women | Range (25-29) | Anxiety and Depression | 165 | EPDS SAS |
| 91 | Yator et al 2021 (188) | Kenya | To assess the preliminary efficacy, acceptability and feasibility of Group Interpersonal Psychotherapy (IP-G) | Primary care | Postpartum women | Median (23.0) Range (18-24) | Depression | 24 | EPDS |
| 92 | Zafar et al 2015 (115) | Malawi | To estimate the extent and distribution of psychological morbidity | Primary care | 1. Pregnant women 2. Postpartum women | Mean (24.5) SD (5.6) | Depression | 1732 | EPDS |
| 93 | Adamu & Adinew 2018 (100) | Ethiopia | To assess the prevalence and factors associated with the symptom of postpartum depression among mothers attending health centres | Primary care (Health Centres) | Postpartum women | Median (28) Inter-quartile range (24–32) | Depression | 629 | EPDS |
| 94 | Oluyemisi & Bawo 2020 (168) | Nigeria | To identify the magnitude of Intimate Partner Violence, and its relationship with psychiatric morbidity and partner alcohol use | Antenatal Clinic of the Central Hospital | Pregnant women | Mean (30.05); SD (5.3) | 1. Depression 2. Anxiety 3. Somatization | 395 | SRQ |
| 95 | Adekanle et al 2015 (169) | Nigeria | To compare psychiatry morbidity among normotensive and hypertensive (cases) pregnant women and their socio-demographic correlates | Primary care  (Antenatal clinic) | Pregnant women | Mean (29.1) SD (5.7) | Depression | 240 | GHQ HADS |
| 96 | Agler et al 2021 (180)a | DRC | To quantify the association of implementing Steps 1–9 or Steps 1–10 on postpartum depressive symptoms and test whether this association was mediated by breastfeeding difficulties | Well baby clinic | Postpartum women | Median (27) | Depression | 903 | EPDS |
| 97 | Atkins et al 2021 (127) | Mozambique | To validate the Alcohol Use Disorder Identification Test (AUDIT) for use in primary care settings | Primary care | 1. Pregnant women 2. Postpartum women | Mean (27.8) SD (7.4) | Alcohol dependence | 533 | AUDIT-10-MZ  MINI 5.0-MZ |
| 98 | Atuhaire et al 2021 (124) | Uganda | To explored lived experiences of women who had recovered from a clinical diagnosis of postpartum depression | Primary care | Postpartum women | Mean (28) | Depression | 30 | DSM-IV |
| 99 | Azale et al 2016 (66) | Ethiopia | To determine the proportion of women with PPD who sought help form a health facility and the associated factors | Primary care | Postpartum women | Mean (28.8) SD (5.2) | Depression | 385 | PHQ-9 GHSQ  SEMI WHODAS |
| 100 | Baron et al 2015 (143) | South Africa | To report the patterns of use of an integrated primary level counselling service among pregnant women | Primary care (Obstetric facility) | Pregnant women | Median (26) | Depression | 3311 | EPDS |
| 101 | Belete & Misgan 2019 (104) | Ethiopia | To assess the prevalence and associated factors of suicidal behaviour (suicidal ideation, plan or suicide attempt) in postpartum mothers | Primary health centres | Postpartum women | Not Stated | Suicidal behaviour | 1065 | MINI AUDIT |
| 102 | Bernsteinn et al 2016 (144) | South Africa | To determine the prevalence and correlates of IPV among HIV-infected pregnant women | Primary care | Pregnant women | Median (28) | 1. Depression 2. Substance use and psychological distress | 623 | EPDS  AUDIT/ DUDIT  K-10 Scale |
| 103 | Chibanda et al 2014 (161) | Zimbabwe | To determine the efficacy of group problem-solving therapy (PST) delivered by peer counsellors versus pharmacotherapy for PND in postpartum HIV-infected and uninfected women attending primary care postnatal clinics | Primary care | Postpartum women | Mean (25) | Depression | 210 | EPDS DSM-IV |
| 104 | Chorwe-Sungani & Chipps 2018 (110) | Malawi | To describe demographic, clinical and risk profile of antenatal depression among pregnant women attending antenatal clinics | Primary care (Antenatal clinics) | Pregnant women | Mean (25.2) SD (5.5) | Depression | 480 | EPDS MINI PRQ |
| 105 | Cumbe et al 2020 (128) | Mozambique | To validate the Patient Health Questionnaire-9 (PHQ-9) for use in primary care settings | Primary care | 1. Pregnant women 2. Postpartum women | Mean (28) | Depression | 502 | PHQ-9 PHQ-2  MINI |
| 106 | Garman et al 2019 (150) | South African | To identify trajectories of perinatal depressive symptoms and their predictors among low-income South African women who were already at risk of depression during pregnancy | Primary care (Community health centres) | Pregnant women | Mean (27)  SD (5.56) | Depression | 384 | HDRS MINI WHODAS AUDIT |
| 107 | Govender et al 2020 (13) | South Africa | To reports on the findings of the prevalence of depression and its associated risk factors among pregnant and postpartum adolescents | Primary care | 1. Pregnant women 2. Postpartum women | Median (18) | Depression | 326 | EPDS |
| 108 | Harrington et al 2018 (111) | Malawi | To estimate the prevalence and incidence of probable perinatal depression among women initiating HIV antiretroviral therapy | Primary care (Antenatal clinic) | Pregnant women | Median (26)  Range (22-30) | Depression | 299 | EPDS  PHQ-9 |
| 109 | Heyningen et al 2019 (145) | South Africa | To develop a psychometrically valid, ultra-short screening tool to detect antenatal depression, anxiety and suicidal ideation | Primary care  (Obstetrics unit) | Pregnant women | Mean (26.9) SD (5.9) | 1. Depression 2. Anxiety | 376 | EPDS  PHQ-9  K-10  Whooley Questions  GAD-2 |
| 110 | Kaaya et al 2010 (17) | Tanzania | To estimate the prevalence of significant depressive symptoms in pregnant women and associations with (i) socio‐demographic and economic status measures; and (ii) quality of relationships with current partners | Primary care | Pregnant women | Mean (25.1) SD (5.8) | Depression | 787 | HSCL |
| 111 | Madeghe et al 2021 (118) | Kenya | To determine the associations between nutrition status, dietary intake, and maternal depression among pregnant women | Primary care (Antenatal clinics) | Pregnant women | Mean (25.3) SD (5.0) Range (18-44) | Depression | 262 | EPDS |
| 112 | Mokhele et al 2019 (146) | South Africa | To measure the prevalence of postpartum depression comparing postpartum HIV-1 infected women with pre-pregnancy HIV care experience, newly diagnosed in latest pregnancy HIV-1 infected women and HIV negative women, and to identify predictors of major postpartum depression among these women in a peri-urban clinic | Primary care (Midwife Obstetric Units) | Postpartum women | median (29) (IQR: 25–33) | Depression | 1151 | CES-D |
| 113 | Mwita et al 2021 (122) | Tanzania | To determine the prevalence and factors associated with depression among pregnant women attending antenatal clinic | Primary care (Antenatal clinic) | Pregnant women | Mean (30.4) | Depression | 345 | EPDS |
| 114 | Peltzer et al 2016 (147) | South Africa | To assess the prevalence of depressive symptoms and associated factors in prenatal HIV-positive women in community healthcare centre | Primary care | Pregnant women | Mean (28.3) SD (5.7) | Depression | 663 | EPDS |
| 115 | Rochat et al 2011 (148) | South Africa | To examine the prevalence of depression and the presentation of depressive symptomatology in antenatal women | Primary care | Pregnant women | Median (24) | Depression | 109 | DSM-IV |
| 116 | Yator et al 2016 (119) | Kenya | To determine the prevalence and severity of postpartum depression (PPD) amongst women living with HIV and to further understand the impact of stigma and other psychosocial factors in women living with HIV attending Prevention of Mother to Child transmission (PMTCT) clinic | Primary care | Postpartum women living with HIV | Mean (31) | Depression | 123 | EPDS |
| 117 | Wemakor & Mensah 2016 (177) | Ghana | To investigate the association between depression among mothers (15–45 years) and stunting in their children (0–59 months) attending Child Welfare Clinic (CWC) | Primary care | Postpartum women | Mean (27.9) SD (8.2) | Depression | 384 | CES-D |
| 118 | Heyningen et al 2018 (137) | South Africa | To address these gaps, compared the psychometric performance of commonly used screening tools to detect Major Depressive Episodes (MDE) and/or anxiety disorders in a sample of low-income women in a resource-poor, primary-level antenatal setting | Primary care (Antenatal clinic) | Pregnant women | Mean (26.8) SD (5.9) | Depression | 376 | EPDS PHQ-9 K-10  K-6 Whooley Questions GAD-2 |
| 119 | Oladeji et al 2022 (170) | Nigeria | To examine the prevalence as well as the clinical and psychosocial factors associated with depression and depression severity in pregnant adolescents from a resource poor setting | Primary Maternal and Child Care | Pregnant adolescents | Mean (18.4); SD (1.0) Range (14-20) Median (19) | Depression | 1359 | EPDS  DSM-IV criteria  CIDI  GAD-7 |

*All primary healthcare settings (formal and non-formal), ** antenatal, pregnant or postpartum women, ******* mean, median or range, ANC-antenatal clinic, DRC= Democratic Republic of Congo,

| **GDAS**: Goldberg Depression and Anxiety Scale | **FTND**: Fagerstrom Test for Nicotine | **PAS**: Psychiatric Assessment Scale |
| --- | --- | --- |
| **PSAS**: Pregnancy-Specific Anxiety Scale | **SRDS**: Self-Rating Depression Scale | **PHQ-2**: Patient Health Questionnaire-2 |
| **GHSQ:** General Help-Seeking Questionnaire | **MINI-KID**: Mini International Neuropsychiatric Interview | **CDI-S**: Child Depression Inventory Short form |
| **SEMI**: Short Explanatory Model Interview | **PSS-19**: Posttraumatic stress symptomology-19 | **CIDI**: Composite International Diagnostic Interview |
| **RCMAS**: Children’s Manifest Anxiety Scale-Revised | **DSQ-19**: Depression Symptom Questionnaire-19 | **HDRS**: Hamilton Depression Rating Scale |
| **OCI-R**: Obsessive Compulsive Inventory-Revised | **PDS:** Posttraumatic Diagnostic Scale | **PSES**: Present State Examination Schedule |
| **CAS:** Clinical Anger Scale | **K-10**: Kessler Psychological Distress Scale | **PSS**: PTSD Symptom Scale |
| **GAD-2**: Generalised Anxiety Disorder Scale-2 | **PRAS:** Pregnancy Related Anxiety Scale | **PRQ:** Pregnancy Risk Questionnaire |
| **WHODAS:** World Health Organization Disability Assessment Tool | **BDI-II:** Becks Depression Inventory II | **SSQ**: Shona Symptom Questionnaire |
| **ASSIST**: Alcohol, Smoking and Substance Involvement Screening Test | **AUDIT:** Alcohol Use Disorder Identification Test | **SAS**: Self-rating Anxiety Scale |
| **GHQ-12**: General Health Questionnaire | **MINI:** Mini International Neuropsychiatric Interview | **CES-D:** Center for Epidemiologic Studies Depression Scale |
| **HADS:** Hospital Anxiety and Depression Scale | **GAD-7**: Generalised Anxiety Disorder Scale-7 | **DSM-IV:** Diagnostic and Statistical Manual of Mental Disorders |
| **PHQ-9**: Patient Health Questionnaire | **HSCL:** Hopkins Symptoms Checklist | **SRQ**: Self-Reporting Questionnaire |
| **EPDS:** Edinburgh Postnatal Depression Scale |  |  |
