# Supplementary material for: Tools for screening maternal mental health conditions in primary care settings in sub-Saharan Africa: systematic review
Source: Front Public Health. 2024 Sep 26;12:1321689. doi: 10.3389/fpubh.2024.1321689 (PMC11466175; doi:10.3389/fpubh.2024.1321689)
Supplement: Supplementary file 5 [file Table_5.docx]

**Table 5: Attributes of screening tools for MMH conditions in primary healthcare facilities in sub-Saharan Africa**

|  |  |  |  |  |  |  | **Attributes of screening tool(s)** | | | | | |
| --- | --- | --- | --- | --- | --- | --- | --- | --- | --- | --- | --- | --- |
| **Ser #** | **Study ID** | **Country** | **Study setting*** | **Participants** | **MMH condition** | **Screening tool (s)** | **Effectiveness** | **Ease of Use** | **Understandability of tool by participants** | **Context relevance** | **Type of health professionals using the tool** | **Adaptability** |
| 1 | Abebe et al 2019 | Ethiopia | Primary care | Postpartum women | Postpartum depression | EPDS | Inferred from outcome of the study | NR | NR | NR | NR | NR |
| 2 | Abiodun 1994 | Nigeria | Primary care in a tertiary setting |  | Anxiety  Depression | 1.HADS 2.GHQ-12 |  | Both screening tools were found to easy and simple to be administered. | NR | The tools were reported to be relevant in identifying probable cases of depression and anxiety among antenatal women | Research assistants whose cadre is not stated | NR |
| 3 | Abiodun 2006 | Nigeria | Primary care | Postpartum women | Postpartum depression | 1. EPDS 2. PSES | The EPDS was shown to be a feasible screening tool for postnatal depression in the PHC centres | The EPDS was reported to be simple to complete | Literate patients self-administered the questionnaire. | NR | A research assistant whose cadre was not stated | The Yuroba version of the EPDS was produced by translation of the questions into the local language. |
| 4 | Abiodun et al 2005 | Nigeria | Primary care | postpartum women | Postpartum depression | EPDS | The screening tool was found to be effective in identifying probable cases of depression | NR | NR | NR | Research assistants whose cadre is not stated. | The Yoruba version of the EPDS was produced through the process of back translation |
| 5 | Abrahams et al 2018 | South Africa | Primary care | Pregnant women | Suicidal ideation and behavior  Depression  Anxiety | MINI-plus | The Expanded MINI Plus tool was found to be effective in identifying depression, anxiety and suicidality. | NR | NR | NR | The MINI was administered by an experienced, registered counsellor, who was supervised by a clinical psychologist | The MINI Plus is available in local South African languages—Afrikaans and isiXhosa . All tools were administered in English, Afrikaans or isiXhosa, the languages spoken by the women. |
| 6 | Abrahams et al, 2019 | South Africa | Primary care | Pregnant women | Depression  Anxiety  Suicidality | EPDS  3-item screening tool | There were identified challenges pertaining to understanding of some of the questions on the EPDS. Despite this, the screening tool was found to be comparable in effectiveness to other studies. | The 3-item too was reported to be easy to administer | Participants had challenges with understanding some of the questions but this improved following adaptation to suit the population  Questions on the 3-item screening tool were well understood | There are some challenges with some of the constructs in the EPDS which had to be modified.  The 3-tem tool was shown to be relevant for use in primary care to screen for depression and can be easily incorporated into routine pregnancy and postpartum care. | First-language English, Afrikaans and isiXhosa speaking women fieldworkers, who had a Bachelor’s degree and professional counselling experience, were trained to seek consent and administer the questionnaires. | English questionnaires (EPDS and 3-item screening tool) and a structured interview guide were forward translated into Afrikaans and isiXhosa by health professionals who were bilingual and familiar with the terminology used in the tools |
| 7 | Acheampong et al 2022 | Ghana | Primary care | Pregnant women | Antenatal depression | PHQ-9 | The validated tool used to measure depression had good psychometric properties | NR | NR | NR | NR | NR |
| 8 | Acheampong et al, 2021 | Ghana | Primary care in a municipality | Pregnant women | Depression | PHQ-9 | The screening tool is reported to be effective in identifying probable cases of depression compared to studies conducted in similar population. | NR | NR | NR | NR | NR |
| 9 | Adamu & Adinew 2018 | Ethiopia | Health Centre | Postpartum women | Postpartum Depression | EPDS | referenced existing literature. | NR | NR | referenced existing literature | diploma nurses, who were not the employees of the selected health centers | NR |
| 10 | Adebowale & James 2020 | Nigeria | Primary care | Antenatal women | Depression  Anxiety  PTSD  Suicidal thoughts | SRQ-20 | Reported the tool was effective in reference to existing literature | Reported the tool was easy to use in reference to existing literature | NR | NR | NR | NR |
| 11 | Adekanle et al 2015 | Nigeria | Primary care in a specialised setting | Antenatal women | Depression | GHQ  HADS | NR | NR | NR | NR | NR | NR |
| 12 | Aderigbedbe & Gureje 1992 | Nigeria | Primary care | pregnant women | 1.Depression 2. Anxiety 3. Phobia | GHQ | The screening tool was found to be effective in identifying depression, anxiety using a revised scoring, | NR | NR | NR | Research assistant whose cadre not specified. | The Yoruba version of the instrument, derived by a modified iterative backtranslation method, |
| 13 | Adeyemo et al, 2020 | Nigeria | Primary Healthcare Centres | Postpartum women | Depression | EPDS | The screening tool is reported to be effective in identifying probable cases of depression compared to studies conducted in similar population. | NR | NR | NR | NR | NR |
| 14 | Agbaje et al, 2019 | Nigeria | Primary Care clinics | Postpartum women | Depression  Anxiety | EPDS  HADS-A | The EPDS and HADS-A are reported to be effective in identifying probable cases of depression compared to studies conducted in similar population. | NR | NR | NR | NR | NR |
| 15 | Agler et al 2021 | DRC | Primary care | Postnatal women | Depression | EPDS | NR | NR | NR | Reference to existing literature that suggests applicability to the study’s context | Healthcare staff whose cadre is not stated. | The original English scale was translated to both French and Lingala by translators fluent in all 3 languages and back translated to ensure fidelity |
| 16 | Alenko et al, 2020 | Ethiopia | Primary care | Pregnant women. | Depression  Substance Use Disorders | BDI-II  ASSIST | The screening tools reported to be effective in identifying probable cases of depression compared to studies conducted in similar population | NR | NR | NR | NR | The English version of BDI-II was translated to Afaan Oromo, and Amharic and finally back translated to English language |
| 17 | Atkins et al 2021 | Mozambique | Primary health care clinics | Antenatal women, postpartum women | Alcohol dependence | AUDIT-10-MZ  MINI 5.0-MZ | In general, the AUDIT-10-MZ and the AUDIT-C-MZ both emerge as valid options to screen for alcohol dependence in Mozambique | found easy to use | found to be comprehensible | the AUDIT-10-MZ had notable variability in the performance of individual questions, with some questions performing poorly in this context | trained data collectors | Given the shortened nature of the AUDIT-C-MZ compared to the full AUDIT-10-MZ, we anticipate it having improved feasibility to be integrated into busy primary care settings with already overburdened primary care staff |
| 18 | Atuhaire et al 2021 | Uganda | Health Centre | Postpartum women | Postpartum depression | DSM-IV | NR | NR | NR | NR | NR | NR |
| 19 | Ayele et al 2016 | Ethiopia | Primary care clinic in a Tertiary setting | Pregnant women | Antenatal depression | BDI | The prevalence of depression could be overestimated due to the presence of somatic symptoms which is normal in pregnancy | NR | NR | NR | Trained nurses working in the ANC clinic collected data | Translated into Amharic and checked for internal constituency by a psychiatrist |
| 20 | Ayele et al, 2021 | Ethiopia | Primary care | Pregnant women | Depression | EPDS | The EPDS is reported to be effective in identifying probable cases of depression compared to studies conducted in similar population | NR | NR | NR | NR | Previously validated EPDS in Ethiopia with the local language was used for this study. |
| 21 | Azale et al 2016 | Ethiopia | Primary care | Postpartum mothers | Postpartum depression | 1. PHQ-9  2. GHSQ  3. SEMI  4. WHODAS | In the latter Ethiopian study of the criterion validity of the PHQ-9, a score of five or more was found to have a sensitivity of 83 % and specificity of 75 % for the detection of major depressive disorder | NR | NR | Reference to existing literature that suggests applicability to the study’s context | Trained data collectors who were trained for 9 days | NR |
| 22 | Bakare et al 2017 | Nigeria | Primary care | Postpartum women | Postpartum depression | EPDS | Not directly commented on. | NR | NR | NR | NR | NR |
| 23 | Baron et al 2015 | South Africa | Primary level obstetric facility | Pregnant women | Antenatal Depression | EPDS | Reported to be effective based on data from published studies | NR | NR | NR | Midwives | Translation and back-translation of the EPDS and RFA were conducted in English, Afrikaans, isiXhosa, |
| 24 | Barthel et al 2015-study 1 | Côte d'Ivoire |  | Pregnant women | Antenatal depression | 1. PHQ-9  2. GAD-7 | PHQ-9 is reported to be effective tool for depression screening if the total score is used. | NR | NR | NR | NR | Translated into a French |
| 25 | Barthel et al 2015-study 2 | Ghana | Primary care in a tertiary setting | Pregnant women | Antenatal depression | 1. PHQ-9  2. GAD-7 | PHQ-9 is reported to be effective tool for depression screening if the total score is used. | NR | NR | NR | NR | Translated into a local language. |
| 26 | Barthel et al 2016-study 1 | Côte d'Ivoire | Primary care | Pregnant women | Antenatal depression  Antenatal and postpartum anxiety | 1. PHQ-9  2. GAD-7 | The PHQ-9 reported to be an effective tool for depression screening if the total score is used. | NR | NR | NR | NR | Tool is reported to have been translated into a French. |
| 27 | Barthel et al 2016-study 2 | Ghana | Primary care in a tertiary setting | Pregnant women | Antenatal depression  Antenatal and postpartum anxiety | 1. PHQ-9  2. GAD-7 | The effectiveness of the GAD-7 scale was based on the psychometric properties of a previously published study that was cited. | NR | NR | NR | The questionnaires were administered by a psychologist with a Master’s Degree | Questionnaires were translated into Twi |
| 28 | Bass et al 2008 | DR Congo | Primary care in a tertiary setting | Postpartum women | Postpartum depression | 1. EPDS  2. HSCL | The alpha scores for the 15-item HSCL, eight-item version of the EPDS screener, and 14-item local screener were 0.86, 0.76 and 0.88 respectively | NR | NR | Context relevant depression-like syndrome from the adapted screening tools and local idioms. | NR | Adaptation of these screeners included translation by using the qualitative data terminology that best reflected the items in the screeners. |
| 29 | Belete & Misgan 2019 | Ethiopia | Primary health centres | Postnatal mothers | Suicidal behaviour | 1.MINI  2. ASSIST | Even though this tool (MINI) was not validated in Ethiopia, a pretest was done and was found to have a good internal consistency (Cronbach’s alpha) of 78% | NR | Though the MINI was not validated in Ethiopia, a pretest was done and was found to be understandable among mothers | Reference to existing literature that suggests applicability to the study’s context | NR | The questionnaire was originally in English, and was translated to Amharic language (local working language). |
| 30 | Bernatsky et al 2007 | Angola | Primary care | Pregnant women | *Poor mental health | GHQ-12 | Reported to be effective in assessing emotional distress from a score of 2 or more. | NR | NR | NR | NR | NR |
| 31 | Bernsteinn et al 2016 | South Africa | Primary Health care antenatal clinic | Pregnant women | Depression  Substance use and psychological distress | 1. EPDS  2. AUDIT/ DUDIT  3. K-10 scale | The SRQ, HSCL-15, EPDS, PRQ and 3-item screener were reliable instruments in the setting (Cronbach’s α= .86, .85, .80, .70 and .70 respectively) | NR | Due to the low literacy levels, the first author and a second research assistant administered the 3-item screener, HSCL-15, SRQ, EPDS and PRQ by reading the questions and recording the answers on behalf of respondents | NR | trained interviewers working in a private room adjacent to the antenatal clinic | The 3-item screener, HSCL-15 and PRQ were translated into Chichewa by the first author and a social worker based on the minimum standards (back translation and monolingual testing) for applying an instrument that was developed in another language |
| 32 | Brittain et al 2017 | South Africa | Primary care | Pregnant women | Antenatal depression | EPDS | Based on previously validated studies used as reference | NR | NR | NR | The EPDS was administered by isiXhosa speaking interviewers due to literacy level concerns. The cadre of the interviewers is not stated. | EPDS was translated into isiXhosa the predominant local language. |
| 33 | Brittain et al, 2015 | South Africa | Primary health care clinics in | Pregnant women | Depression | BDI-II | The screening tool is reported to be effective in identifying probable cases of depression compared to studies conducted in similar population | NR | NR | NR | NR | NR |
| 34 | Brittain et al, 2019 | South Africa | Antenatal Clinic | Pregnant women | Depression | EPDS | Reported to be effective in identifying probable cases of depression compared to studies conducted in similar population | NR | NR | NR | Interviewers whose cadre is not indicated. | Study measures were administered by trained interviewers in isiXhosa, the predominant local language |
| 35 | Chibanda et al 2010 | Zimbabwe | Primary care | Postpartum women | Postpartum depression | EPDS | The Shona version of the EPDS was found to be effective and comparable to other validation studies with similar population characteristics. | NR | All participants were able to comprehend the questions on the EPDS. | The version used was found to be context relevant. | Community counselors | Translated into the Shona language culturally relevant terminology for depression |
| 36 | Chibanda et al 2014 | Zimbabwe | Primary care clinics | Postpartum women | Depression | EPDS  DSM-IV | NR | NR | NR | EPDS reported by previous studies to suit the local context | Trained primary care peer counselors  DSM-IV was used by psychiatrists | NR |
| 37 | Chorwe & Chipps 2018 | Malawi | Primary care | Pregnant women | Depression | 1. EPDS  2. HSCL  3. SRQ  4. PRQ  5. 3-item screener | With the exception of the 3-item screener, there was no difference in performance in identifying probable cases of depression among pregnant women HSCL-15, SRQ were found to be comparable what is found in literature. | NR | NR | NR | Nurse and two midwives | Previously validated Chichewa language versions of the EPDS and the SRQ existed and were used in this study. The 3-item screener, HSCL-15 and PRQ were translated into Chichewa by the first author and a social worker based on the minimum standards |
| 38 | Chorwe-Sungani & Chipps, 2018b | Malawi | Antenatal clinics | Pregnant women | Depression | 1. 3-item Screener  2. HSCL  3. EPDS  4. SRQ  5. MINI |  |  |  |  |  |  |
| 39 | Chorwe-Sungani & Chipps 2018 | Malawi | Antenatal clinics | Antenatal Women | Depression | 1. EPDS  2. MINI  3. PRQ | Consistent with previous studies,13 our findings showed that the EPDS remains to be a valid instrument (sensitivity of 68.0%, specificity of 88.0% and AUC = 0.85) for detecting antenatal depression when used in its original form locally | NR | The interviewer administration of screening instruments may have influenced respondents to give answers that they deemed as socially acceptable in the presence of the interviewer | NR | Research assistants trained in administration of the EPDS and PRQ collected the data from 480 pregnant women | The PRQ and the MINI were translated into Chichewa by the researcher and a bilingual social worker through forward and backward translations |
| 40 | Cumbe et al 2020 | Mozambique | Primary healthcare clinics | Pregnant and Postpartum women | Depression | 1. PHQ-9  2. PHQ-2  3. MINI | NR | NR | The PHQ-MZ instrument was found to be comprehensible, appropriate and easy to understand | found to be relevant | trained data collectors under supervision | Both PHQ and MINI adapted to the Mozambique context |
| 41 | Cyimana et al 2010 | Zambia | Primary care in a tertiary setting | Postpartum women | Postpartum depression | EPDS | Although prevalence of depression was found to be comparable that reported in other studies, the data was not validated. | NR | NR | NR | Research assistant whose cadre in the healthcare profession is not indicated. | NR |
| 42 | Dadi et al 2020 | Ethiopia | Primary care in secondary and tertiary settings | Pregnant women | Depression | EPDS | The effectiveness of the screening tool is based on the psychometric properties established in literature | NR | NR | NR | Nurses | NR |
| 43 | Dlamini et al 2019 | Eswatini  (Swaziland) | Primary care | Postpartum women | Postpartum depression | EPDS | The reported performance of the tool is based on previous validated studies. | NR | NR | NR | NR | NR |
| 44 | E-Andjafono et al 2020* | Congo | Primary care | Postpartum women | Maternal depression | 1. EPDS 2. GDAS  3. DSM-IV (MINI) Criteria | NR | NR | NR | NR | NR | NR |
| 45 | Emmerson et al 2020 | Congo |  | Postpartum women | Anxiety  Depression  PTSD | 1. HSCL-25 | The effectiveness of HSCL-25 is confirmed by the validity of a Swahili version of the scale has been evaluated in a sample of Tanzanian women using content and construct validation methods | NR | NR | NR | NR | The questionnaire was administered in Swahili, the predominant local language |
| 46 | Fantahun et al, 2018 | Ethiopia | Primary Healthcare | Postpartum women | Depression | EPDS | The effectiveness of the EPDS based generated sensitivity and specificity of 78.9% and 75.3% respectively from a previous validation study. | NR | NR | NR | Diploma nurses who were not employees of the selected health centers collected data | NR |
| 47 | Garman et al 2019 | South African | Community health centres | Antenatal women | Depression | 1. HDRS  2. MINI  3. WHODAS  4. AUDIT | NR | NR | NR | NR | Trained community health workers | NR |
| 48 | Govender et al 2020 | South Africa | Primary care (rural clinic) | Antenatal and Postpartum women | Depression | EPDS | NR | NR | NR | NR | Trained fieldworkers from the community | NR |
| 49 | Green et al 2018 | Kenya | Primary care | Pregnant and postpartum women | Perinatal depression | 1. EPDS  2. PHQ-9  3. PDEPS | The EPDS and PHQ-9 are valid and reliable screening tools for perinatal depression in rural Western Kenya, but a new 9-item locally-developed tool called the Perinatal Depression Screening (PDEPS) that blends Western psychiatric concepts and local idioms of distress may be a more useful alternative | NR | NR | NR | BSc level Kenyan enumerators with a background in mental health or social work. | NR |
| 50 | Guo et al 2014-study 1 | Côte d’Ivoire | Primary care | Pregnant and postpartum women | 1. Perinatal Depression  2. Perinatal Anxiety | 1. PHQ-9  2. GAD-7 | The reliability of the PHQ-9 and GAD-7 was found to be moderate which impairs the precision of the measurements | NR | Proposed different understanding of the wording of the questionnaires particularly the GAD-7 (predominantly used in high income countries). | NR | NR | Questionnaires translated into French, the official language and adapted for administration by an interviewer. |
| 51 | Guo et al 2014-study 2 | Ghana | Primary care in a tertiary setting | Pregnant and postpartum women | 1. Perinatal. Depression  2. Perinatal Anxiety | 1.PHQ-9  2. GAD-7 | The reliability of the PHQ-9 and GAD-7 was found to be moderate which impairs the precision of the measurements | NR | Proposed different understanding of the wording of the questionnaires particularly the GAD-7 (predominantly used in high income countries). | NR | NR | Questionnaires translated into Twi the dominant local language in Ghana and adapted for administration by an interviewer. |
| 52 | Gureje et al, 2019 | Nigeria | Primary maternal clinics | Pregnant and postpartum women | Depression | EPDS | The effectiveness of the EPDS based generated previous work. | NR | NR | NR | Research assistants whose cadre is not stated. | The EPDS to those women who consented to be screened and spoke Yoruba, the language of the study. |
| 53 | Gureje et al, 2019b | Nigeria | Primary maternal clinics | Pregnant and postpartum women | Depression | PHQ-9 | The effectiveness of the EPDS based on previous work. | NR | NR | NR | Research assistants whose cadre is not stated | The PHQ-9 questionnaire was delivery in Yoruba |
| 54 | Harrington et al 2018 | Malawi | Primary care | Pregnant care | Antenatal depression | EPDS | Effectiveness of the screening tools based on comparison to reported values from literature. | NR | Questions may not have been understood by study participants. | NR | Study nurses | NR |
| 55 | Harrington et al 2018 | Malawi | Antenatal clinic | Perinatal Mothers | Depression | 1. EPDS  2. PHQ-9 | The EPDS and PHQ-9 scores had high concordance when using the dichotomous cut-off points | NR | The EPDS and the PHQ-9 may not be adequately capturing the burden of probable perinatal depression in our population due to imperfect translation of words or concepts into the Chichewa versions. “Depression” itself may mean something different to women in Malawi, and may manifest in ways outside of the scope of the EPDS or PHQ-9 | NR | Nurses | NR |
| 56 | Heyningen et al 2018 | South Africa | Primary care antenatal clinic | Antenatal Women | Depression  Anxiety | 1.EPDS  2. EPDS-3A  3. PHQ-9  4. PHQ-2  5. K-10 scale  6. K-6 scale  7. Whooley questions  8. Whooley + help  9. GAD-2 | The K10, K6, PHQ9, and Whooley questions (with the help question) showed good internal consistency, while the EPDS, Whooley questions (without the help question), and GAD-2 had slightly lower consistency. The EPDS performed poorly when conducted in isiXhosa compared to other languages. In detecting MDE, all tools performed well. For anxiety disorders, K10 and K6 had the highest accuracy. For both MDE and anxiety, EPDS, K10, K6, PHQ9, and Whooley questions (with help question) had high accuracy, while EPDS (3-item), PHQ2, and Whooley questions (without help question) showed slightly lower accuracy | Whooley questions performed the best. Furthermore, the psychometric performance of the two Whooley questions is comparable to the 10-item EPDS, the K10 and the 9-item PHQ9. In addition to being ultra-short, it is a simple binary-scoring instrument that is possibly more feasible and acceptable for busy, low-resource primary care settings for use by non-specialist health workers than longer, Likert-type tools | NR | In this setting, the EPDS and K6 were the best performing tools to detect MDE | This was conducted by a field-worker with an honours degree in Psychology and four years of experience as a research assistant in clinical settings. The second stage comprised a structured diagnostic interview carried out by a registered counsellor, with a Bachelor of Psychology (Honours) degree in counselling. Both were trained and supervised by a clinical psychologist | NR |
| 57 | Heyningen et al 2019 | South Africa | primary care | Antenatal women | 1.Depression  2.Anxiety | 1. EPDS  2. PHQ-9  3. K-10 scale  4. Whooley questions  5. GAD-7  6. GAD-2 | The main finding of this study is that symptoms of MDE and anxiety and suicidal ideation in low-resource settings can be detected using an ultra-short, binary-scoring screening tool. The performance of this tool is comparable to longer screening tools and has several advantages  The sensitivity (78%) and specificity (82%) of our tool in detecting MDE and anxiety disorders seems favourable compared to the EPDS (75%; 78%) and the PHQ9 (66%; 76%), as well as ultrashort versions of these: the 3-item EPDS (70%; 77%), the PHQ2 (75%; 69%) and the Whooley questions (66%; 87%). | In order to standardise the scoring system, certain Likert-type scoring items were adapted to be binary scoring. Although this was done for ease of use in clinical application, this may have affected the accuracy of the scoring | NR | NR | A research assistant and mental health officer were appointed to collect data and provide counselling | There is also potential to adapt and test the tool for mobile technology platforms, where it can be self- administered, thereby providing an assessment of mental health problems outside of clinical settings. |
| 58 | Heyningen et al,2018 | South Africa | Primary care | Pregnant women | Depression and Anxiety | EPDS  K10  K6  PHQ-9  Whooley | The EPDS was the best performing instrument for detecting MDE and the K10 and K6 for anxiety disorder. For MDE and/or anxiety disorders, the EPDS had the highest AUC (0.83). Of the short instruments, the K10 (AUC = 0.85) and the K6 (AUC = 0.85) performed the best, with the K6 showing good balance between sensitivity (74%) and specificity (85%) and a good positive predictive value (70%). | NR | NR | The relevance of these screening tools in non-research settings not assessed. | This was conducted by a field-worker with an honours degree in Psychology and four years of experience as a research assistant in clinical setting | The field-worker administered all the English and isiXhosa and Afrikaans. |
| 59 | Ikeako et al 2018 | Nigeria | Postnatal clinic | Postpartum women | Depression | EPDS | The screening tool reported to be effective in identifying probable cases of depression compared to studies conducted in similar population | NR | NR | NR | The questionnaires were administered by a trained female member of the research team | NR |
| 60 | January & Chimbari 2018 | Zimbabwe | Primary care | Postpartum women | Postpartum depression | 1. EPDS  2. PHQ-9  3. CES-D | The effectiveness of the screening tools was in reference to other studies. | NR | NR | NR | Degree level research assistants whose cadre was not stated | NR |
| 61 | January et al 2015 | Zimbabwe | Primary care | Postpartum women | Postnatal depression | EPDS | The Shona version of the EPDS is a simple, valid and effective screening tool for detection of postnatal depression amongst HIV infected and uninfected postnatal m others in Zimbabwe | NR | NR | .NR | 4th year medical students | The validated Shona version of the Edinburgh Postnatal Depression Scale (EPDS) was used for data collection |
| 62 | Kaaya et al 2008 | Tanzania | Primary care | Pregnant women | Depression | 1. HSCL-25  2. DSQ-19 | The effectiveness of the screening tools was found to be effective in identifying depression. | NR | NR | NR | Trained research assistants whose cadre is not indicated. | The Kiswahili version was translated, back-translated and refined as described in another article. |
| 63 | Kaaya et al 2010 | Tanzania | Primary Health Care clinics | Antenatal women | Depression | HSCL | NR | NR | NR | NR | NR | Assessment of current depressive symptoms at recruitment was by face‐to‐face interviews with a Kiswahili adapted version of the Hopkins Symptom Checklist (KHSCL) |
| 64 | Kakyo et al 2012 | Uganda | Primary care | Postpartum women | Postpartum depression | EPDS | Effectiveness of the screening tools based on comparison to reported values from literature | NR | NR | The EPDS does not extensively address the somatic manifestations of depression which in the African population have been reported to be a common manifestation of postpartum depression. | researchers (Mental Health Nurse, Psychiatric Clinical Officer and Social Worker) | NR |
| 65 | Kimbui et al 2018 | Kenya | Primary care | Pregnant women | Depression  Substance use | 1. AUDIT  2. EPDS  3. BDI II | The EPDS is reported to be effective in identifying probable cases of depression as indicated by the validation studies.  The BDI II demonstrated high internal consistency. The study reported the BDI (II) as a viable and reliable measure for identifying probable cases of Depressive disorders among adolescents. | The self-report version of the AUDIT which was used is reported to be easy to and takes less than 5 min to complete | NR | NR | NR | The Kiswahili translated version of the EPDS was used. |
| 66 | Kugbey et al 2021 | Ghana | Primary care | Pregnant women | Antenatal Depression  Anxiety  Suicidal behaviours | HADS | Reported to be effective in screening for depression in pregnant women. | NR | NR | NR | Cadre of staff administering the tool not indicated. | NR |
| 67 | MacGinty et al 2020 | South Africa | Primary care | Pregnant women | Antenatal psychological distress  Antenatal depression, and PTSD | 1.SRQ-20  2. BDI | Psychological Distress and depression reported to be comparable with that found in literature | NR | NR | NR | Questionnaires were administered to participants but their cadre is not reported. | Questions were read to participants in English, Africans or isiXhosa based on the participant preference. |
| 68 | Madeghe et al 2021 | Kenya | Antenatal care clinics | Antenatal women | Depression | EPDS | NR | NR | NR | NR | NR | NR |
| 69 | Mahenge et al 2013 | Tanzania | Primary care | Pregnant women | PTSD  Anxiety  Depression | 1. HSCL 25  2. PDS | The HSCL-25 has been validated in Tanzania however the PTSD scale though not validated locally had been validated in Uganda (cited as reference measurement for this study) | NR | NR | NR | Medical doctor | the questionnaires were translated into Kiswahili |
| 70 | Mahenge et al 2015 | Tanzania | Primary care | Pregnant women | PTSD  Anxiety  Depression | 1. PDS  2. HSCL-25 | The HSCL-25 has been validated in Tanzania however the PTSD scale though not validated locally had been validated in Uganda (cited as reference measurement for this study) | NR | NR | NR | Research assistants whose cadre is not indicated | Both scales were translated into Swahili |
| 71 | Mahenge et al2018 | Tanzania | Primary care | Postpartum women | Postpartum depression | PHQ-9 | The effectiveness of the PHQ-9 is based on validation studies done in Tanzania | NR | NR | NR | NR | NR |
| 72 | Malemela & Mashegoane 2019 | South Africa | Primary care | Pregnant women | Antenatal depression  Anxiety  Anger during pregnancy  OCD | 1. EPDS  2. OCI-R  3. PRAS  4. CAS | The effectiveness of the screening tools is based on the psychometric properties established in literature | NR | NR | NR | NR | NR |
| 73 | Mandell et al 2021 | South Africa | Primary care | Pregnant women | Depression  Suicidal ideation | EPDS | In this study, Cronbach’s alpha for the EPDS was σ=0.81, suggesting good internal consistency as an indication of probable depression. | NR | NR | NR | NR | NR |
| 74 | Manikkam and Burns 2012 | South Africa | Primary care | Pregnant women | Antenatal depression | EPDS | The scale has been validated in South Africa previously a satisfactory sensitivity (79%) | NR | NR | NR | Self-Administered | Translated into Zulu however it was completed in English or isiZulu and completed by the participant. |
| 75 | Mebrahtu et al 2018 | Zimbabwe | Primary care | Postpartum women | Maternal stress  Maternal depression | 1. EPDS  2. PSI-SF | The effectiveness of the EPDS is based on validation studies from literature. | NR | NR | NR | NR | NR |
| 76 | Mokhele et al 2019 | South Africa | Primary care (Obstetric unit) | Postpartum mothers | Depression | CES-D 10 | NR | NR | NR | NR | NR | NR |
| 77 | Mokwena and Masike 2020 | South Africa | Primary care | Postpartum women | Postpartum depression | EPDS | Reported effectiveness is based on previous validated studies using the EPDS. | NR | NR | NR | Researcher and research assistants whose cadre is not reported | NR |
| 78 | Mossie et al 2017 | Ethiopia | Primary care | Pregnant women | Antenatal depression | BDI | The screening tool is reported to be effective in identifying probable cases of depression compared to studies conducted in similar population. | NR | NR | NR | Psychiatric Nurse | The questionnaire was translated from English to a local language (Tigrigna) and back to English by professional translators with the assistance of a mental health specialist. |
| 79 | Mwita et al 2021 | Tanzania | Primary care | Antenatal Women | Depression | EPDS | NR | NR | NR | NR | NR | NR |
| 80 | Nakku et al, 2006 | Uganda | Primary health care centre | Postpartum Women | Depression | 1. SRQ-25,  2. MINI | The SRQ was reported to be effective in identifying probable cases of depression compared to studies conducted in similar population | NR | NR | NR | Doctor (House Officer) | All questionnaires had been independently translated and back translated to and from Luganda |
| 81 | Necho et al 2020 | Ethiopia | Primary care | Postpartum women | Postpartum depression | EPDS | NR | NR | NR | NR | BSc Nurses administered the questionnaire | NR |
| 82 | Nhiwatiwa et al 1998 | Zimbabwe | Primary care | Pregnant women followed-up to 8 weeks postpartum | Postnatal mental Disorders | SSQ | The validity data of this questionnaire show that a cut of score of 7 of 8 has the optimal balance of sensitivity and specificity for case detection | NR | NR | The SSQ is principally based on local idioms and was found to be acceptable to women;  the risk of confounding caused by the somatic experiences associated with pregnancy was considered by the interviewers to be minimal. | Village Health workers | NR |
| 83 | Nyamukoho et al 2019 | Zimbabwe | Primary care | Pregnant women | Depression | EPDS | NR | NR | NR | NR | Research assistants whose cadre is not stated | NR |
| 84 | Nydoo et al 2017 | South Africa | Primary care | Pregnant women | Depression | EPDS | The effectiveness of the EPDS is based on studies from literature. | NR | NR | NR | Nurse | The EPDS was translated into an isiZulu version for the purpose of this study. |
| 85 | Odinka et al 2018 | Nigeria | Primary care in a tertiary setting | Postpartum women | Postpartum depression  Postpartum anxiety | HADS | Its effectiveness in screening and case-finding is based on previously published literature. | NR | Very minimal assistance was given to the mothers as they were literate enough to complete the questionnaires. | NR | Self-administered by the participants | NR |
| 86 | Okeke 2021 | Nigeria | Primary care | Pregnant women followed-up to postpartum | Postpartum depression | EPDS | The screening tool is reported to be effective in identifying probable cases of depression compared to studies conducted in similar population. | NR | NR | NR | NR | Translation of the EPDS was done by a local translator |
| 87 | Oladeji et al 2022 | Nigeria | Primary Maternal and Child Care | Pregnant women (adolescents) | Depression | 1.EPDS  2. GAD-7 | NR | NR | NR | NR | NR | NR |
| 88 | Ongeri et al 2018 | Kenya | Primary care | Pregnant women | Perinatal and postpartum depression | EPDS | NR | NR | NR | NR | Nurses | NR |
| 89 | Osok et al, 2018 | Kenya | Primary care facility | Pregnant adolescents | Depression | 1.PHQ-9,  2.EPDS | The screening tools reported to be effective in identifying probable cases of depression compared to studies conducted in similar population | NR | NR | NR | Clinical Psychologist | The Kiswahili version of the EPDS and PHQ-9 were used. |
| 90 | Peltzer 2011 | South Africa | Primary care | Postpartum women living with HIV | Postpartum depression | EPDS | The effectiveness of the screening tool (EPDS) is based on the psychometric properties previous studies in South Africa. |  |  | NR | Trained interviewer whose cadre is not specified. | The EPDS was adapted to isiXhosa version |
| 91 | Peltzer et al, 2016 | South Africa | Community Health Centres | Antenatal women | Depression | EPDS | Cronbach alpha for the EDPS-10 scale was 0.80 in this study sample, showing adequate internal consistency | NR | NR | NR | NR | The Edinburgh Postnatal Depression Scale 10 was used to assess depression, adapted for perinatal depression in English, Zulu or Sotho |
| 92 | Peltzer et al, 2018 | South Africa | Community health centres | Pregnant and postpartum HIV infected women | Depression | EPDS | The effectiveness of the EPDS based generated previous work. | NR | NR | NR | NR | NR |
| 93 | Phukuta & Omole 2020 | South Africa | Primary care i | Postpartum women | Postpartum Depression | EPDS | The EPDS was found to be effective in identifying probable cases of depression and importantly mothers who were suicidal or had intents of harming their babies as result of the underlying condition. | NR | NR | NR | Doctors and research assistants whose care is not stated. | NR |
| 94 | Robert et al 2022 | South Africa | Primary care | Pregnant adolescents/ women | Depression  PTSD  Suicidal symptoms  Anxiety | 1. CDI-S  2. MINI-KID  3. RCMAS  4. PTSD checklist | The effectiveness of the screening tools is based on the psychometric properties established in literature | NR | NR | NR | NR | All the questionnaires were translated into isiXhosa for participants to complete in the language of their choice (isiXhosa or English) |
| 95 | Rochat et al 2011 | South Africa | Primary health clinic | Antenatal Women | Depression | DSM-IV | NR | NR | Women used psychological language in their description of their experience of depression and depressed women were able to make the subtle distinctions between metaphorical and culturally specific descriptions and their depressive symptoms | NR | Two South African IsiZulu-speaking researchers were trained to interview mothers using the major depression section of the structured clinical interview for DSM-IV diagnoses | NR |
| 96 | Rochat et al, 2013 | South Africa | Primary Health Care facility | Pregnant women | Depression | 1. EPDS (3-item) | The performance of the short ultrashort EPDSR3 version supports evidence that shorter version of the EPDS may be effective in detecting antenatal depression | Reported to be “User friendly” | NR | The study reports that the rate of depression was higher on the clinical interview method than on the EPDS screening measure implying probable cases of depression could potentially be missed in the target population | NR | The women were interviewed in the local language (Zulu) |
| 97 | Sorsdahl et al, 2015 | South Africa | Primary healthcare in Cape Town | Pregnant women | Depression  Substance use disorders | 1. EPDS  2. FTAD  3. ASSIST | NR | NR | NR | NR | Trained nurse | NR |
| 98 | Spedding et al, 2020 | South Africa | Primary health centre | Pregnant women | Depression  Psychological Distress | 1. EPDS  2. SRQ-20  3. ASSIST  4. PSS | NR | NR | NR | NR | Intake nurses were trained to use the EPDS | NR |
| 99 | Stellenberg et al 2015 | South Africa | Primary care | Postpartum women | Postnatal depression | 1. EPDS  2. BDI | The reported effectiveness is based on previously published studies. | NR | Some questions (items) were not readily understood by participants | NR | Researcher/trained field worker with a nursing background | All instruments were pre-tested and rectified, where required. |
| 100 | Stewart et al 2009 | Malawi | Primary care | Postpartum women | Maternal depression | SRQ | This Chichewa version of the SRQ shows utility as a brief screening measure for detection of probable maternal depression in rural Malawi | Can be administered quickly (time not stated) | NR | Can be used consistently by non-health workers | A field worker whose cadre is not reported on. | The SRQ was independently translated into Chichewa by 4 bilingual Malawians: a psychiatrist , a clinical psychologist , a psychiatric nurse and a secondary school teacher |
| 101 | Stewart et al 2013 | Malawi | Primary care | Pregnant women | Antenatal depression | 1. EPDS  2. SRQ  3. DSM-IV | The criterion validation showed that both EPDS and SRQ had satisfactory test characteristics as screening measures for depressive disorder in an antenatal population. | The interviewers found both the SRQ and EPDS straightforward to administer.  The authors report they improved local ease of use through simplification of the answers between items and use of a visual prompt card, although this may lessen the technical equivalence with the original version | Both EPDS and SRQ were acceptable to respondents and regarded as straightforward to administer by the interviewers, although the need to first explain the visual prompt card added complexity to the EPDS in comparison with the SRQ. | NR | Research assistants whose cadre is not stated | Translated and modified in Chichewa language. |
| 102 | Stewart et al 2014 | Malawi | Primary care | Antenatal women | Antenatal depression | 1. SRQ  2. EPDS | Validated Chichewa and Chiyao versions of two depression screening tools were administered. | NR | NR | NR | The fieldworkers were trained Chichewa- and Chiyao-speaking female school leavers | Interviews were conducted in either Chichewa or Chiyao, depending on the participant’s preference |
| 103 | Tesfaye and Agenagnew et al, 2021 | Ethiopia | Primary Healthcare centre | Pregnant women | Depression | PHQ-9 | NR | NR | NR | NR | Trained midwives | NR |
| 104 | Tungchama et 2016 | Nigeria | Primary care | Postpartum women | Postpartum depression | 1. EPDS  2. DSM-IV | The effectiveness is based on the psychometric properties of the tool established by other researchers in the Nigerian context. | NR | NR | NR | NR | NR |
| 105 | Tuthill et al, 2017 | South Africa | Primary healthcare facilities | Pregnant and postpartum HIV positive women | Depression | PHQ-9 | NR | NR | NR | NR | trained research assistants | NR |
| 106 | Ukaegbe et al 2012 | Nigeria | Primary care in a secondary setting | Postpartum women | Postpartum depression | EPDS | The effectiveness of the EPDS is based on studies from literature | NR | NR | NR | NR | The EPDS was translated in Igbo. |
| 107 | Umuziga et al 2015 | Rwanda | Primary care | Perinatal women | Perinatal depression  Perinatal anxiety | 1. SAS  2. EPDS | The effectiveness of the screening tools is based on studies from literature | NR | NR | NR | NR | NR |
| 108 | Umuziga et al, 2020 | Rwanda | Primary Health Centres | Pregnant and postpartum women | Depression and Anxiety | 1.EPDS  2. SAS | These standardized scales used were found to be reliable; the Cronbach’s alpha was 0.89 and 0.87 for EPDS and SAS respectively | NR | NR | NR | NR | NR |
| **109** | **Uwakwe 2003** | Nigeria | Primary care | Postpartum women | Affective morbidity | 1. EPDS  2. SRDS | The EPDS can thus effectively discriminate between depressed and non-depressed postnatal women.  Both the EPDS and SRDS showed good internal consistency | The EPDS will have high utility in the busy postnatal clinics, considering that it is short and relatively easy to administer | NR | The EPDS was found to be relevant for the context in assessing postpartum depression in Nigerian women. | Resident doctors in Obstetrics and Gynaecology. | The two scales were translated into the Igbo language. |
| 110 | Van Heyningen et al 2016 | South Africa | Primary care | Pregnant women | Common Perinatal Mental Disorders (CPMD) | **MINI Plus** | The MINI Plus has been validated in a previous study in South Africa whose psychometric properties the current study refers to. | NR | NR | NR | Mental Health Officer | The MINI Plus was translated into isiXhosa and Afrikaans |
| 111 | Wemakor & Mensah 2016 | Ghana | Primary care | Postpartum women | Depression | CES-D | CES-D has not been validated in Ghana and therefore, no clinical threshold has been established to distinguish between those depressed and not depressed and its reliability in this context is unknown | NR | NR | NR | The administration of the questionnaire was undertaken by two final year undergraduate students of the School of Medicine and Health Sciences, UDS, Tamale. | The questionnaire was administered in English in a face-to-face interview with the mothers, and for those who did not speak English it was translated and administered in Akan or Dagbani language |
| 112 | Woldetensay et al 2018 | Ethiopia | Primary care | Pregnant women | Depression | 1. PHQ-9  2. MINI | The PHQ-9 was effective at identifying a probable case of depression in the sample population with a sensitivity at a cut of ≥8 score was 80.8% with specificity of 79.5% | NR | The cognitive debriefing revealed that the PHQ-9 was generally well understood. | The cognitive debriefing revealed that the PHQ-9 was generally acceptable and culturally appropriate for all the respondents | Nurse | Translation and cultural adaptation of the PHQ-9 was performed  The depression component of the MINI Plus was translated into Afaan Oromo language. |
| 113 | Wong et al 2017 | South Africa | Primary care | Pregnant women | Antenatal depression | EPDS | The effectiveness of the EPDS is based on studies from literature | NR | NR | NR | NR | NR |
| 114 | Wubetu et al 2020 | Ethiopia | Primary care in a tertiary setting | Postpartum women | Postpartum depression | EPDS | The effectiveness of the EPDS was in reference to comparative studies in other settings. No validation was done in the current study | NR | NR | NR | Undergraduate Nursing students | NR |
| 115 | Yamamoto et al 2020 | Tanzania | Primary care in a tertiary setting | Pregnant women | 1. Stress 2. Anxiety | PRAS-revised | The effectiveness of the two screening tools is based on the psychometric properties established in literature. | NR | NR | NR | NR | Both the PRAS-revised and PSS tools were translated into Swahili. |
| 116 | Yator et al 2016 | Kenya | Primary care in a tertiary setting | Postpartum women living with HIV | Depression | EPDS | NR | NR | NR | NR | NR | NR |
| 117 | Yator et al 2021 | Kenya | Postnatal clinic | Postpartum women | Depression | EPDS | NR | NR | NR | NR | Community health workers | NR |
| 118 | Zafar et al,2015 | Malawi | Primary healthcare facility | Pregnant and postpartum women | Depression | EPDS | The EPDS performed well with Cronbach’s reliability alpha coefficient of 0.71 in Malawi | nurse-midwives found the questions and assessments easy to carry out as these were on the whole similar to those used during routine antenatal or postnatal care in both setting | Women found the questions easy to answer and non-threatening | NR | nurse-midwives | NR |
| 119 | Zelalem et al 2020 | Ethiopia | Primary care | Postpartum depression | Antenatal depression | EPDS | The effectiveness of the EPDS is based on studies from literature | NR | NR | NR | Midwives | NR |

| **GDAS**: Goldberg Depression and Anxiety Scale | **FTND**: Fagerstrom Test for Nicotine | **PAS**: Psychiatric Assessment Scale |
| --- | --- | --- |
| **PSAS**: Pregnancy-Specific Anxiety Scale | **SRDS**: Self-Rating Depression Scale | **PHQ-2**: Patient Health Questionnaire-2 |
| **GHSQ:** General Help-Seeking Questionnaire | **MINI-KID**: Mini International Neuropsychiatric Interview | **CDI-S**: Child Depression Inventory Short form |
| **SEMI**: Short Explanatory Model Interview | **PSS-19**: Posttraumatic stress symptomology-19 | **CIDI**: Composite International Diagnostic Interview |
| **RCMAS**: Children’s Manifest Anxiety Scale-Revised | **DSQ-19**: Depression Symptom Questionnaire-19 | **HDRS**: Hamilton Depression Rating Scale |
| **OCI-R**: Obsessive Compulsive Inventory-Revised | **PDS:** Posttraumatic Diagnostic Scale | **PSES**: Present State Examination Schedule |
| **CAS:** Clinical Anger Scale | **K-10**: Kessler Psychological Distress Scale | **PSS**: PTSD Symptom Scale |
| **GAD-2**: Generalised Anxiety Disorder Scale-2 | **PRAS:** Pregnancy Related Anxiety Scale | **PRQ:** Pregnancy Risk Questionnaire |
| **WHODAS:** World Health Organization Disability Assessment Tool | **BDI-II:** Becks Depression Inventory II | **SSQ**: Shona Symptom Questionnaire |
| **ASSIST**: Alcohol, Smoking and Substance Involvement Screening Test | **AUDIT:** Alcohol Use Disorder Identification Test | **SAS**: Self-rating Anxiety Scale |
| **GHQ-12**: General Health Questionnaire | **MINI:** Mini International Neuropsychiatric Interview | **CES-D:** Center for Epidemiologic Studies Depression Scale |
| **HADS:** Hospital Anxiety and Depression Scale | **GAD-7**: Generalised Anxiety Disorder Scale-7 | **DSM-IV:** Diagnostic and Statistical Manual of Mental Disorders |
| **PHQ-9**: Patient Health Questionnaire | **HSCL:** Hopkins Symptoms Checklist | **SRQ**: Self-Reporting Questionnaire |
| **EPDS:** Edinburgh Postnatal Depression Scale |  |  |
